# BOULE is Essential for the Dynamic Disassembly of Heat Shock Granules in Male Germ Cells

**DOI:** 10.1002/advs.77155

**Published:** 2026-08-13

**Authors:** Xin Li, Nana Zhang, Zexin Bian, Baobao Geng, Liyun Cao, Peipei Liu, Zengming Li, Yan Zhao, Jun Tan

**Affiliations:** ^1^ Reproductive Medicine Center Jiangxi Maternal and Child Health Hospital Nanchang Medical College Nanchang Jiangxi Province China; ^2^ Center For Reproductive Medicine The Third Affiliated Hospital of Zhengzhou University Zhengzhou Henan Province China; ^3^ State Key Laboratory of Reproductive Medicine and Offspring Health Nanjing Medical University Nanjing Jiangsu China; ^4^ State Key Laboratory of Common Mechanism Research for Major Diseases Suzhou Institute of Systems Medicine Chinese Academy of Medical Sciences & Peking Union Medical College Suzhou Jiangsu China

**Keywords:** BOULE, G3BP1, germ cell, heat shock, stress granule

## Abstract

Heat shock triggers stress granule (SG) formation, yet their dynamics in mammalian germ cells remain unclear. Through systematic analysis, we uncovered distinct heat shock granule (HSG) composition and stage‐specific assembly/disassembly in the mouse testis. Ribosome profiling revealed that germ cells selectively sustain translation of spermatogenic genes during heat shock. We identified that the germ cell‐specific RNA‐binding protein BOULE is essential for HSG clearance. BOULE deficiency under heat shock impairs HSG disassembly, with increased germ cell apoptosis and a disrupted ubiquitinome. Mechanistically, BOULE orchestrates HSG clearance through a two‐pronged mechanism. It post‐transcriptionally maintains the expression of the disassembly factors G3BP1 and FAF2. Additionally, BOULE promotes heat‐shock‐induced ubiquitination of G3BP1 by regulating the E3 ligase TRIM27, thereby recruiting the VCP/FAF2 complex. Our findings establish BOULE as a key regulator of proteostasis that coordinates HSG disassembly in germ cells, providing mechanistic insight into heat shock‐induced germ cell damage that may contribute to male infertility.

## Graphical abstract

BOULE orchestrates stress granule disassembly in germ cells via a two‐pronged mechanism: it promotes G3BP1 ubiquitination by upregulating TRIM27, generating a signal for VCP/FAF2 recruitment, and it maintains G3BP1 and FAF2 protein levels. BOULE deficiency disrupts disassembly complex formation, leading to impaired heat shock granule clearance.

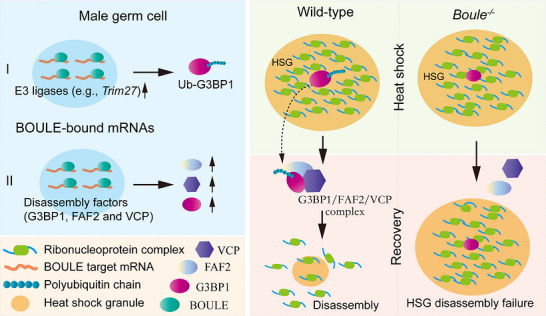

## 1 Introduction

Stress granules (SGs) are dynamic, membraneless organelles formed via liquid–liquid phase separation of low‐complexity protein domains in the cytoplasm under various stress conditions, including heat shock, oxidative stress, arsenite (As), osmosis (Os), starvation, and viral infection [1, 2, 3, 4, 5, 6, 7]. These stress granules are primarily composed of RNA‐binding proteins, mRNAs, and translational machinery [1, 2, 8, 9]. They play crucial roles in translational reprogramming and cellular adaptation. The assembly and disassembly dynamics of stress granules in somatic cells are well established, a process governed by RNA‐binding proteins (e.g., G3BPs), protein interaction networks, molecular chaperones, the ubiquitin‐proteasome system, autophagy pathways, and ATP‐dependent factors such as VCP/p97 [1, 3, 5, 10, 11, 12, 13, 14, 15, 16, 17, 18, 19, 20, 21, 22, 23]. While SGs were traditionally viewed as translationally silent compartments, recent single‐molecule imaging studies have challenged this notion by demonstrating that mRNAs within SGs can still be translated [9, 24, 25], suggesting that SGs may function as dynamic mRNA management hubs rather than simple translational repressors. Persistent or aberrant SG formation is closely linked to neurodegenerative diseases, cancer, and multisystem proteinopathies [11, 26, 27, 28, 29, 30, 31, 32, 33], highlighting the importance of regulated SG dynamics in both cellular stress adaptation and pathogenesis.

Compared to somatic cells, germ cells undergo unique and complex differentiation processes such as meiosis, chromatin remodeling, and spermiogenesis [34, 35, 36], which may necessitate cell type‐specific stress response mechanisms. Germ cells are particularly sensitive to environmental stressors, and heat shock is known to impair spermatogenesis and cause male infertility [19, 37, 38, 39, 40]. Although evidence suggests that germ cell‐specific RNA‐binding proteins, such as DAZL and BOULE [41, 42], are enriched in HSGs and contribute to their functions, whether spermatogenic HSGs possess unique core protein components and how these components regulate HSG assembly and disassembly dynamics remain poorly understood. Furthermore, the global impact of heat shock on the translatome of germ cells and its relationship with HSG dynamics remain a key unresolved question. Understanding the mechanisms governing HSG dynamics in germ cells is essential for deciphering how spermatogenesis adapts to environmental stress and for elucidating the molecular basis of stress‐related male infertility.

Recent advances in proteomics, translatomics, single‐cell transcriptomics, and super‐resolution imaging have enabled systematic characterization of the composition and dynamics of ribonucleoprotein (RNP) granules. In this study, we integrated multi‐omics approaches with functional validation to investigate the assembly and disassembly mechanisms of HSGs in testicular germ cells under heat shock. By optimizing HSG purification strategies, we systematically identified the proteomic and RNA compositions of heat shock granules in the testis. We further delineated the stage‐specific dynamics of HSG assembly and disassembly during spermatogenesis. Moreover, we identified a unique regulatory mechanism in germ cells wherein the RNA‐binding protein BOULE orchestrates HSG disassembly through two parallel pathways: promoting G3BP1 ubiquitination by upregulating the E3 ligase TRIM27, and post‐transcriptionally controlling G3BP1 and FAF2 expression. These dual pathways cooperate to promote the assembly of the functional G3BP1/VCP/FAF2 disassembly complex. Collectively, our findings define a novel regulatory paradigm for HSGs in the germline and advance the understanding of stress‐responsive proteostasis in male reproductive biology.

## 2 Results

### 2.1 Identification of Germ Cell‐Specific Core Protein Components in Heat Shock Granules

**FIGURE 1:**
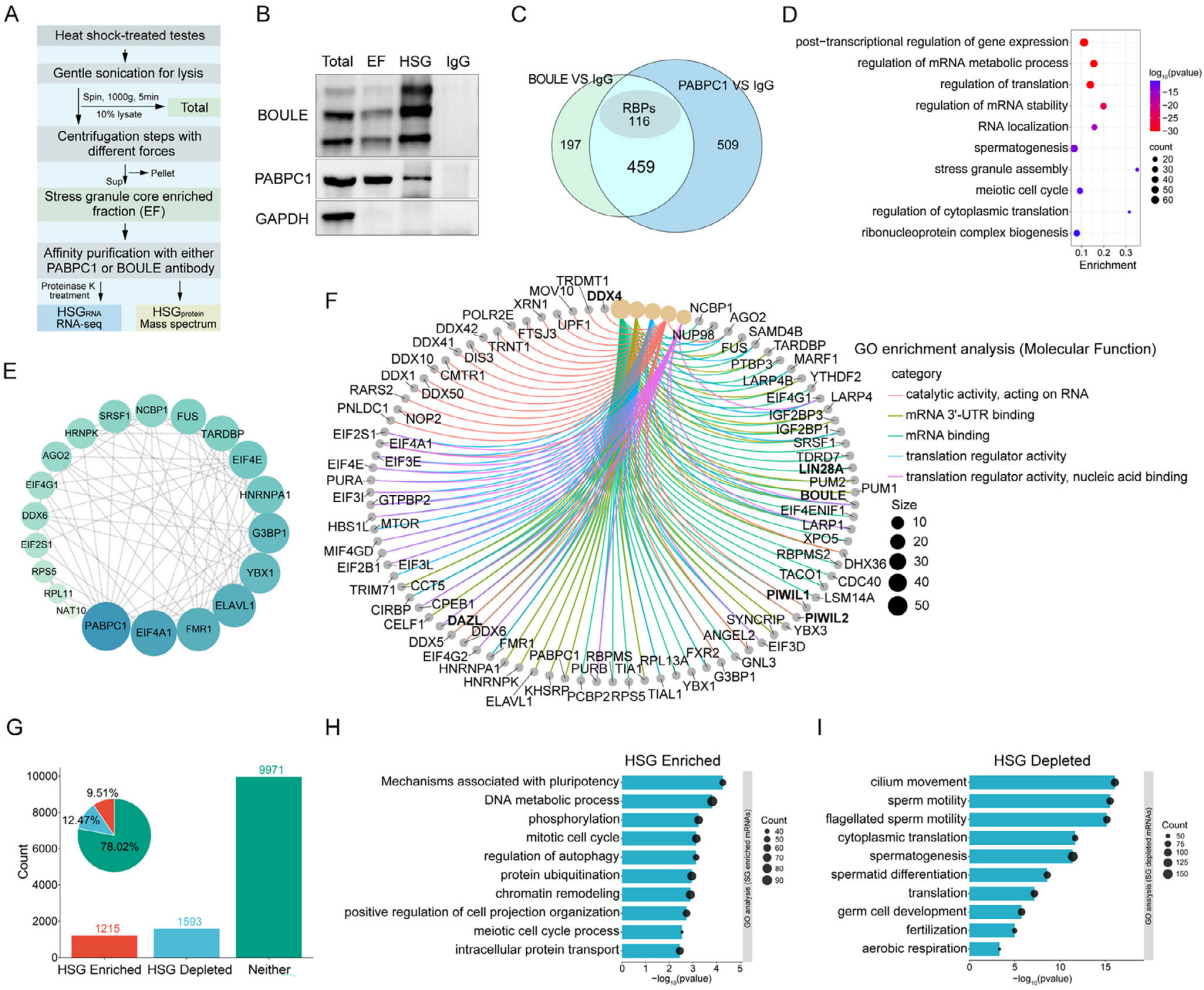
Proteomic and transcriptomic profiling of stress granule (SG) cores in murine male germ cells. (A) Schematic workflow for HSG core purification from mouse testis after heat shock. (B) Western blot analysis of BOULE, PABPC1, and GAPDH in total testicular lysate (Total), the HSG core‐enriched fraction (EF), and purified HSG cores (HSG). IgG serves as a negative control. (C) Immunopurification and proteomic identification of HSG core components. Venn diagram showing numbers of proteins identified by anti‐BOULE and anti‐PABPC1 pulldowns (IgG as background control). RBPs, RNA‐binding proteins. (D) Gene Ontology (GO) enrichment analysis of biological processes for the 459 proteins identified in the testicular HSG core fraction. (E) Protein–protein interaction (PPI) network analysis of the identified HSG core proteome. The PPI network was generated using the STRING database and visualized with Cytoscape. The top 20 hub proteins, ranked by connectivity, are highlighted. (F) GO molecular function analysis of the HSG core proteome. A concept network plot illustrates the top five enriched molecular function terms and their associated proteins. Proteins in bold black font are specifically expressed in germ cells. (G) Transcriptome analysis of mRNAs associated with germ cell HSG cores. HSG cores were immunopurified using anti‐BOULE or anti‐PABPC1 antibodies, with total testicular RNA as a control. The bar graph shows the overlap of genes identified under different conditions between BOULE‐ and PABPC1‐purified samples. The adjacent pie chart depicts the proportion of mRNA species categorized as: “HSG enriched” (*FC* > 2, *p* < 0.05), “HSG depleted” (*FC* < 0.5, *p* < 0.05), or “Neither” (no significant change). (H) GO biological process enrichment analysis for mRNAs significantly enriched in HSG cores. (I) GO biological process enrichment analysis for mRNAs significantly depleted from SG cores.

To systematically characterize the protein and RNA composition of heat shock granules in testicular germ cells, we adapted established methods for somatic SG isolation [43, 44, 45] to purify testicular HSGs (Figure 1A), followed by mass spectrometry (MS) and RNA‐seq. HSG cores were enriched based on two distinct markers: the canonical SG protein PABPC1 and the germ cell‐specific protein BOULE, respectively. Western blot analysis confirmed significant enrichment of both PABPC1 and BOULE in HSG core‐enriched fractions and purified HSG cores (Figure 1B), validating successful HSG core isolation. Mass spectrometry identified 656 proteins from BOULE‐enriched HSG cores and 968 proteins from PABPC1‐enriched HSG cores (Figure 1C). By integrating these datasets, we identified 459 germ cell‐specific HSG proteins, including 116 RNA‐binding proteins (Figure 1C, Data S1,S2). These proteins are significantly enriched in GO pathways primarily associated with post‐transcriptional regulation of gene expression, regulation of mRNA metabolic process, regulation of translation, spermatogenesis, stress granule assembly, and meiotic cell cycle (Figure 1D). This profile is similar to the composition of somatic stress granules [15], with the most notable distinction being the involvement of spermatogenesis‐related proteins. This highlights the unique core functionality of heat shock granule proteins specific to germ cells. Protein–protein interaction network analysis identified the top 20 hub proteins, including known SG components [15, 43] such as PABPC1, EIF4A1, FMR1, ELAVL1, YBX1, and G3BP1 (Figure 1E; Figure S1), suggesting a shared molecular basis between germ cell and somatic SGs. Further molecular function GO analysis highlighted the top five pathways concentrated in RNA binding and translational regulation (Figure 1F). A protein‐pathway network map indicated that among 117 proteins, 6 were specifically expressed in testicular germ cells, including DAZL, BOULE, DDX4, LIN28A, PIWIL1, and PIWIL2 (Figure 1F). These germ cell‐specific HSG components likely constitute the unique molecular foundation of germ cell HSGs.

### 2.2 Transcriptomic Profiling Reveals a Functional Bias in mRNA Enrichment During Heat Shock

We next performed RNA sequencing on HSG cores purified via PABPC1 and BOULE immunoprecipitation. A total of 12 779 genes with FPKM > 1 in total RNA were included in the analysis. We identified 1215 transcripts (9.51%) significantly enriched in HSGs, a proportion similar to the 9.4% reported in somatic cells [44], and 1593 transcripts (12.47%) depleted from HSGs (Figure 1G). Functional enrichment analysis revealed that HSG‐enriched genes were primarily associated with mechanisms associated with pluripotency, DNA metabolic processes, phosphorylation, mitotic cell cycle, and regulation of autophagy (Figure 1H). Notably, HSG‐depleted genes were enriched in pathways related to cilium movement, sperm motility, flagellated sperm motility, cytoplasmic translation, and spermatogenesis (Figure 1I). Given that poorly translated transcripts are typically enriched in HSGs, whereas highly translated mRNAs are excluded, our findings suggest that spermatogenesis‐related genes may not undergo translational repression under heat shock, but rather maintain or enhance their translational activity in germ cells.

### 2.3 Stage‐Specific Assembly of Heat Shock Granules in Male Germ Cells With Peak Sensitivity in Early Pachytene Spermatocytes

**FIGURE 2:**
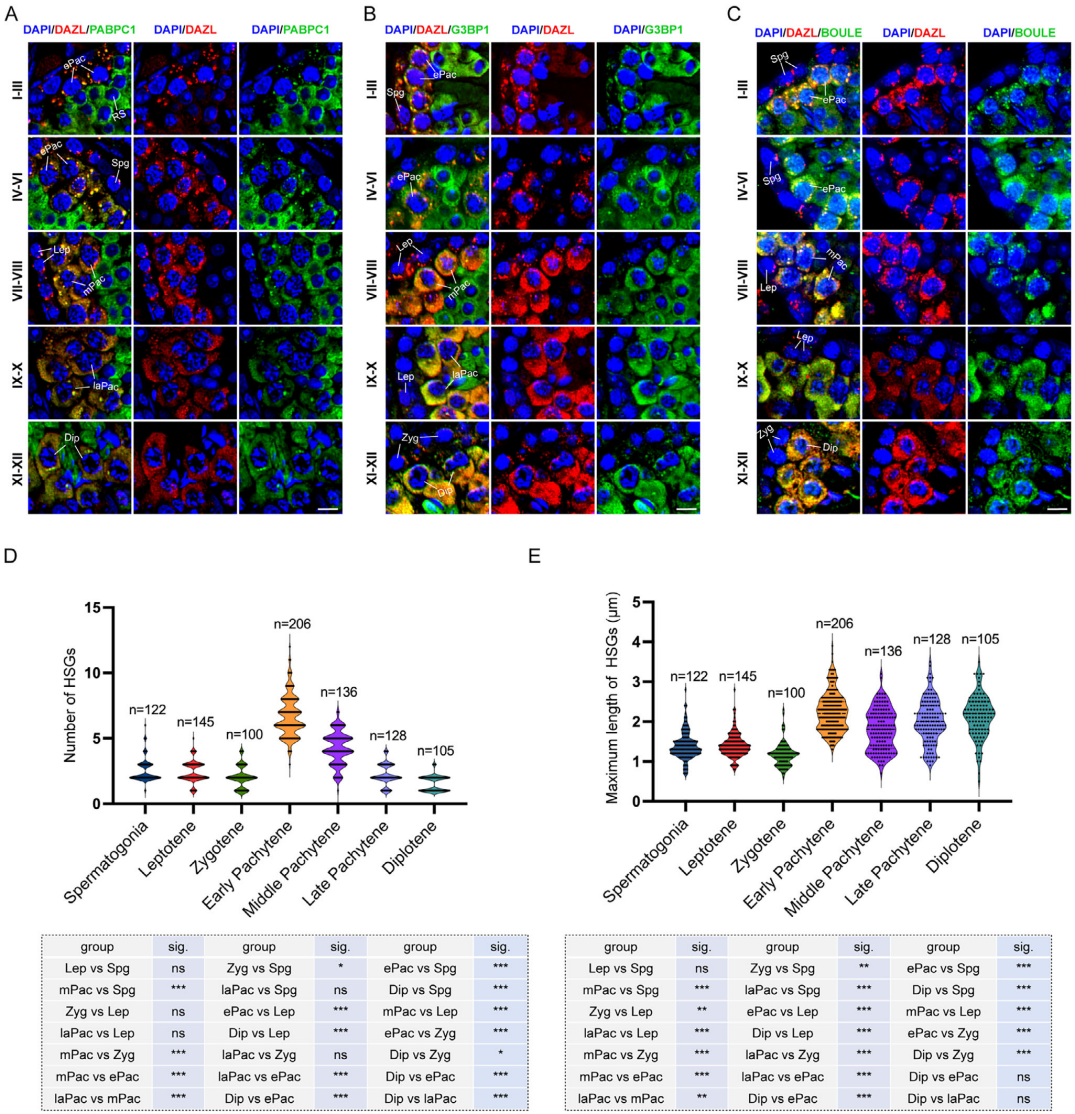
Quantitative analysis of heat shock‐induced stress granules across germ cell stages reveals stage‐specific differences in granule number and size. Representative immunofluorescence images of testis sections showing co‐localization of germ cell and stress granule markers across distinct stages of the seminiferous epithelial cycle. Stages were classified (I–III, IV–VI, VII–VIII, IX–X, and XI–XII) based on luminal cell composition and cytological architecture. (A) Co‐staining for the germ cell marker DAZL (red) and the stress granule core marker PABPC1 (green). (B) Co‐staining for DAZL (red) and the stress granule marker G3BP1 (green). (C) Co‐staining for DAZL (red) and BOULE (green). Nuclei are counterstained with DAPI (blue). Scale bars: 10 µm. (D) The number of SGs per germ cell was quantified and compared between different developmental stages. (E) The maximum diameter of SGs in germ cells was measured and compared across stages. Data are presented as mean ± SD. N represents the number of germ cells counted. Statistical significance was assessed using one‐way ANOVA followed by Tukey's post hoc test. *, **, and *** indicate *p* < 0.05, *p* < 0.01, and *p* < 0.001, respectively; ns indicates not significant. Spg, spermatogonia; Lep, leptotene; Zyg, zygotene; ePac, early pachytene; mPac, middle pachytene; laPac, late pachytene; Dip, diplotene; RS, round spermatid.

To investigate the dynamic assembly and disassembly of heat shock granules in germ cells, we performed immunofluorescence staining using four established heat shock granule markers (DAZL, PABPC1, G3BP1, and BOULE) [15, 41, 42] to characterize heat shock granules across different developmental stages. Under control conditions, all four proteins exhibited diffuse cytoplasmic localization in germ cells (Figures S2–S4). Using established seminiferous tubule staging criteria [46] and integrating the expression patterns of these markers, we classified germ cells into their respective developmental stages. Under the experimental conditions of 42°C for 30 min of heat shock, round spermatids consistently failed to form detectable stress granules, as assessed by multiple SG markers including DAZL, PABPC1, G3BP1 and BOULE, whereas at all other developmental stages of germ cells, prominent heat shock granules were readily observed (Figure 2A–C). Our results showed that zygotene spermatocytes had lower levels of heat shock granules, both in number and size, compared to other developmental stages (Figure 2D,E). In contrast, pachytene spermatocytes, particularly those at early and mid‐pachytene stages, exhibited robust granule‐forming capacity, with early pachytene spermatocytes displaying the highest number and largest size of heat shock granules (Figure 2D,E). Despite having fewer heat shock granules (Figure 2D), late pachytene and diplotene spermatocytes possessed stress granules of relatively large size compared to other stages (Figure 2E). These findings indicate that the highly stage‐specific assembly of heat shock granules in male germ cells, with early pachytene spermatocytes exhibiting heightened sensitivity to heat shock, may represent an active adaptive strategy to safeguard critical meiotic processes such as homologous recombination and DNA repair.

### 2.4 Developmental Stage‐Specific Dynamic Disassembly of Heat Shock Granules in Germ Cells

**FIGURE 3:**
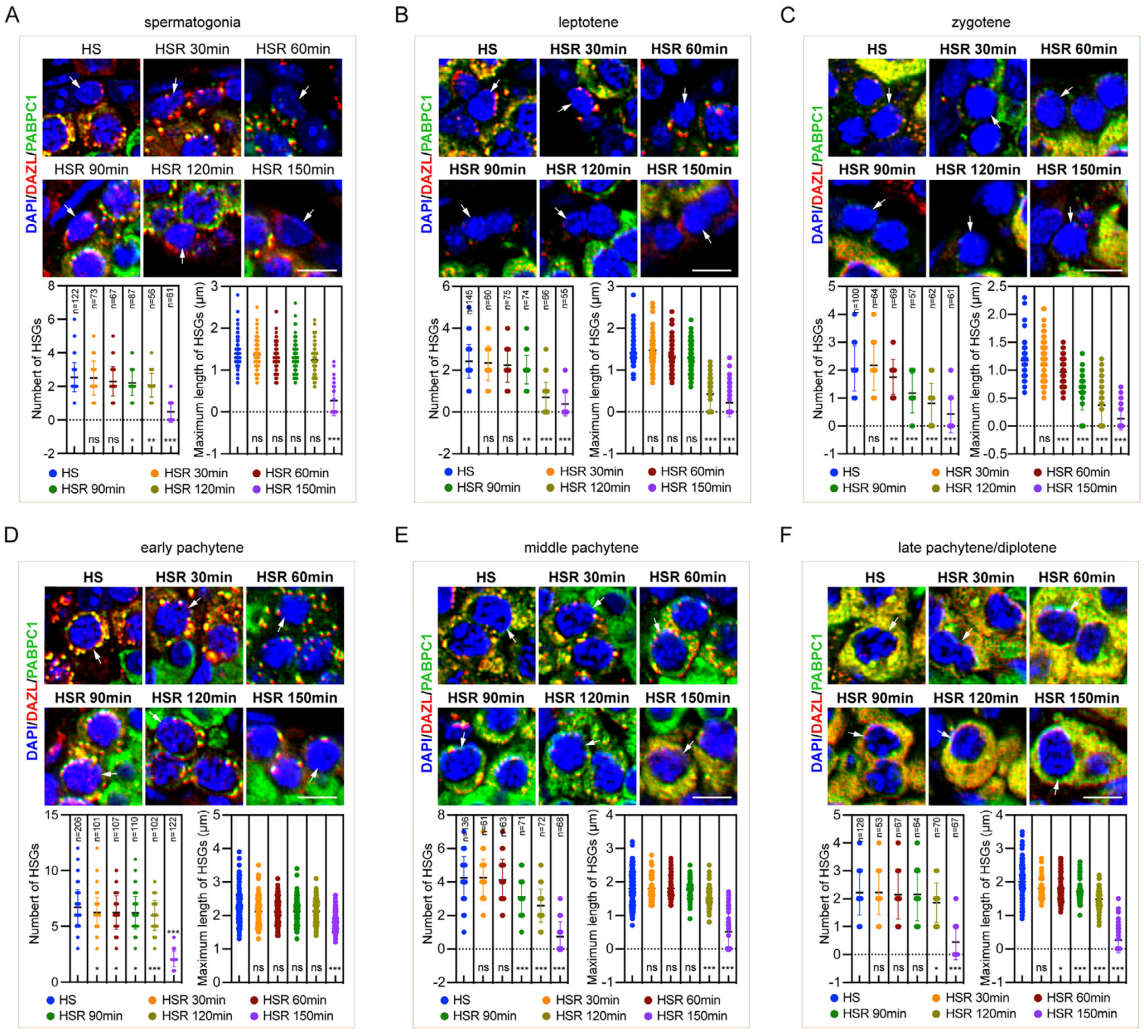
Dynamic assembly and disassembly of heat shock granules in stage‐specific germ cells during heat shock and recovery. Co‐immunofluorescence staining for DAZL (red) and PABPC1 (green) was performed to label heat shock granules in testicular sections. Nuclei were counterstained with DAPI (blue). White arrowheads indicate the nuclei of stage‐specific germ cells. (A–F) Representative immunofluorescence images (upper panels) and corresponding quantitative analysis (lower panels) of stress granule abundance (left graphs) and maximum diameter (right graphs) in specific germ cell types under heat shock (HS) and recovery (HSR) conditions. Stages analyzed include: (A) spermatogonia. (B) leptotene spermatocytes. (C) zygotene spermatocytes. (D) early pachytene spermatocytes. (E) mid‐pachytene spermatocytes. (F) late pachytene/diplotene spermatocytes. Note that for several panels, the same fluorescence micrograph was used to highlight different cell types within the same proximal field of view. Quantification data are presented as mean ± SD. Statistical significance was determined using one‐way ANOVA followed by Tukey's post hoc test. ns, not significant; *, *p* < 0.05; **, *p* < 0.01; ***, *p *< 0.001. *n* = number of cells counted for each group. Scale bar: 10 µm.

To delineate the stage‐specific dynamics of heat shock granule disassembly in the male germline, we performed multiplex immunofluorescence using antibodies against germ cell‐specific HSG markers, namely DAZL and BOULE, as well as core stress granule components including PABPC1 and G3BP1 (Figure 3; Figures S5,S6). Germ cells were analyzed at sequential post‐heat‐shock recovery time points of 30, 60, 90, 120, and 150 min under room temperature (RT) conditions. HSGs were quantified based on their number and maximum diameter.

Our analysis revealed distinct disassembly kinetics across germ cell subtypes. Strikingly, early pachytene spermatocytes exhibited a pronounced and rapid disassembly of HSGs, characterized by a rapid reduction in the number of heat shock granules within the first 30 min of recovery, whereas granule size remained stable throughout the 120‐min recovery period (Figure 3D). In contrast, significant disassembly of heat shock granules was not observed in spermatogonia (Figure 3A), leptotene spermatocytes (Figure 3B), or mid‐pachytene spermatocytes (Figure 3E) until 90 min of recovery. Zygotene spermatocytes displayed intermediate disassembly kinetics, with a significant decrease in SG parameters at 60 min post‐recovery (Figure 3C). Intriguingly, the dynamics of SG number and size diverged in late pachytene and diplotene spermatocytes (Figure 3F). While SG counts persisted unchanged until 120 min, a marked decrease in SG size commenced from the 30‐min time point onward (Figure 3F). However, from spermatogonia to mid‐pachytene spermatocytes, a consistent feature was that the number of heat shock granules changed prior to their size (Figure 3A–E). A pivotal finding was the synchronous and sharp decline in SG number and size across all germ cell types at the 150‐min recovery, marking the onset of a cross‐cell‐type clearance phase that ensures tissue‐wide restoration of homeostasis (Figure 3). Collectively, these data demonstrate that germ cells employ stage‐specific strategies to resolve cytoplasmic stress, reflecting the distinct functional properties of germ cells and the underlying regulatory principles governing their adaptation to external stimuli.

### 2.5 Dynamic Translatome Reprogramming During Heat Shock and Recovery

While stress granule formation is known to cause a dramatic reduction in translation in somatic cells [1], its global effect on translation in germ cells under heat shock remains unclear. To address this, we performed ribosome profiling sequencing (Ribo‐seq) on mouse testes subjected to heat shock and subsequent recovery, generating the first dynamic translatome atlas for the testis throughout the heat shock (HS) response and recovery (HSR). Given the observed disassembly of heat shock‐induced granules in all germ cell types by 150 min of recovery (Figure 3), we selected this time point to investigate the reactivation of translation.

**FIGURE 4:**
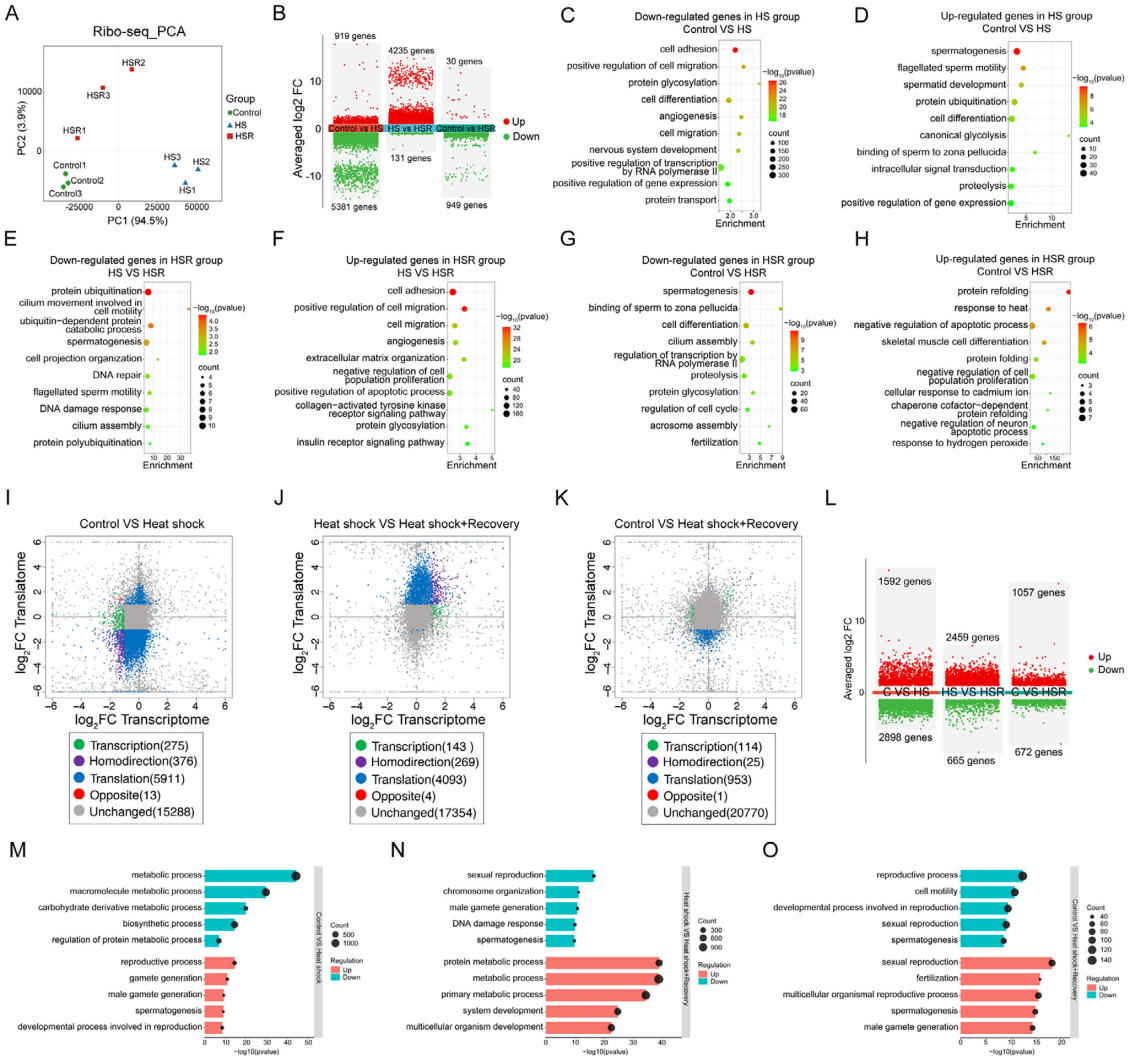
Characterization of translatome dynamics throughout testicular heat shock and recovery. (A) Principal component analysis (PCA) of samples based on translatome expression profiles, illustrating the distance relationships between groups. HS, heat shock; HSR, heat shock followed by 2.5 h of recovery. (B) Volcano plots showing significantly differentially expressed genes (DEGs) among the three groups. The *X*‐axis represents pairwise comparisons between groups, and the *Y*‐axis indicates the avg_log2FC (average log_2_ fold change). Bubble chart showing gene ontology (GO) enrichment analysis for translationally down‐regulated genes (C) and translationally up‐regulated genes (D) in the HS condition (Control vs. HS comparison). Bubble chart showing GO enrichment analysis for translationally down‐regulated genes (E) and translationally up‐regulated genes (F) in the HSR condition (HS vs. HSR comparison). Bubble chart showing GO enrichment analysis for translationally down‐regulated genes (G) and translationally up‐regulated genes (H) in the HSR condition (Control vs. HSR comparison). For panels C─H, the *X*‐axis represents the gene ratio, the *Y*‐axis indicates the enriched GO terms, the color scale corresponds to different *p*‐value thresholds, and the dot size reflects the number of genes associated with each pathway. (I–K) Comparative analysis of the translatome and transcriptome across different group comparisons. Genes are categorized into five classes based on their differential expression thresholds in the two omics datasets (log2FC ≥ 1 and *FDR* < 0.05 for significantly upregulated genes; log2FC ≤ −1 and *FDR* < 0.05 for significantly downregulated genes). The categories are defined as follows: Transcription, genes significant only in the transcriptome; Translation, genes significant only in the translatome; Homodirection, genes significant in both omics with concordant regulation direction (both upregulated or both downregulated); Opposite, genes significant in both omics but with discordant regulation direction; Unchanged, genes with no significant change in either omics. (L) Volcano plot analysis of genes with significantly altered translation efficiency (TE) across groups. TE is calculated as the ratio of ribosome‐bound (actively translating) mRNA to total mRNA for each gene. Each pairwise comparison is labeled in the plot. Genes with significantly increased TE are marked in red, and those with significantly decreased TE are marked in green. C, control; HS, heat shock; HSR, heat shock followed by recovery. (M–O) GO enrichment analysis of genes with differential translation efficiency for the control versus HS (M), HS versus HSR (N), and control versus HSR (O) comparisons.

We first validated the quality of our Ribo‐seq data by confirming the characteristic “high‐low‐low” three‐nucleotide periodicity of the obtained ribosome‐protected fragments (RPFs) (Figure S7A). The number of genes with mapped RPFs was substantially reduced upon heat shock, but largely returned to control levels after recovery (Figure S7B), indicating that the heat shock‐induced suppression of translation is largely reversible. Consistently, both the genomic distribution of RPFs and gene read‐coverage ratios were markedly decreased under HS and recovered toward baseline in HSR samples (Figure S7C,D). Principal component analysis (PCA) revealed distinct clustering of the HS group from both the Control and HSR groups (Figure 4A). Collectively, these results demonstrate that heat shock profoundly alters translational activity in the testis.

We next characterized the dynamic translational reprogramming during heat shock and recovery. Comparative analysis revealed 5381 genes with significantly decreased translation in HS testes compared to controls (Figure 4B; Figure S7E). These genes were enriched in pathways related to cell adhesion, positive regulation of cell migration, and cell differentiation (Figure 4C), suggesting a diminished capacity for cellular remodeling in the testis upon stress. Interestingly, 919 genes were translationally upregulated during heat shock (Figure 4B). These upregulated genes were significantly associated with pathways including spermatogenesis, flagellated sperm motility, and spermatid development (Figure 4D), indicating a selective maintenance of core germ cell functions, which aligns closely with our findings from the transcriptomic analysis of heat shock granules (Figure 1I). The upregulation of the protein ubiquitination pathway at the translational level is consistent with the observed increase in cellular ubiquitination following heat shock in somatic cells [15] (Figure 4D). During the recovery phase, translation was upregulated for 4235 genes (Figure 4B; Figure S7F), which significantly overlapped with the genes downregulated during heat shock (Figure 4F), while translation was downregulated for 131 genes (Figure 4B), primarily those associated with spermatogenesis (Figure 4E). However, when compared directly to the unstressed control, only 30 genes were translationally upregulated (Figure 4B,H), while 949 genes were downregulated (Figure 4B; Figure S7G). These downregulated genes were primarily linked to spermatogenesis (Figure 4G). Thus, we observed a selective translational prioritization of spermatogenic genes during acute heat shock, which dissipated upon stress removal.

### 2.6 Widespread Reprogramming of Translational Efficiency Drives Testicular Heat Shock Adaptation and Recovery

Parallel bulk RNA‐seq analysis showed high Pearson correlation coefficients among all groups (Figure S8A). Differential expression analysis identified 646 differentially expressed genes (DEGs) between control and HS groups, 416 DEGs between HS and HSR groups, and 140 DEGs between control and HSR groups (Figure S8B). The functional annotations of these gene sets are presented in Figure S8C–E. In contrast to the massive reprogramming of translation, transcriptional alterations remained relatively modest under heat shock. A direct comparison between changes at the transcriptome and translatome further confirmed that heat shock and recovery predominantly affect gene translation rather than transcription (Figure 4I–K).

To directly assess translation regulation independent of mRNA abundance, we calculated translational efficiency (TE) for each gene. Heat shock significantly altered the TE of 4490 genes compared to the control, with 1592 upregulated and 2898 downregulated (Figure 4L). Genes with decreased TE were enriched in pathways such as metabolic process, macromolecule metabolic process, and carbohydrate derivative metabolic process (Figure 4M). Conversely, genes with increased TE were predominantly associated with spermatogenesis (Figure 4M). Following SG disassembly (HSR vs. HS), the TE of 2459 genes increased while that of 665 genes decreased (Figure 4L). TE‐elevated genes were related to metabolism and development, whereas TE‐reduced genes were linked to spermatogenesis (Figure 4N). Notably, even after the cycle of stress and recovery (HSR vs. Control), 1729 genes exhibited TE alterations (Figure 4L). Both increased and decreased TE gene sets in this comparison were significantly enriched for spermatogenesis (Figure 4O). In summary, translational efficiency analysis suggests a proposed model of translational prioritization during acute heat stress: heat shock prioritizes spermatogenic translation over metabolism, a pattern that reverses during recovery. However, persistent TE dysregulation specifically in spermatogenic genes is hypothesized to represent a lasting molecular disturbance that may underlie heat shock‐induced germ cell apoptosis, though this remains a working hypothesis rather than a proven causal mechanism.

### 2.7 A Single‐Cell Transcriptional Atlas of the Testicular Response to Acute Heat Shock

**FIGURE 5:**
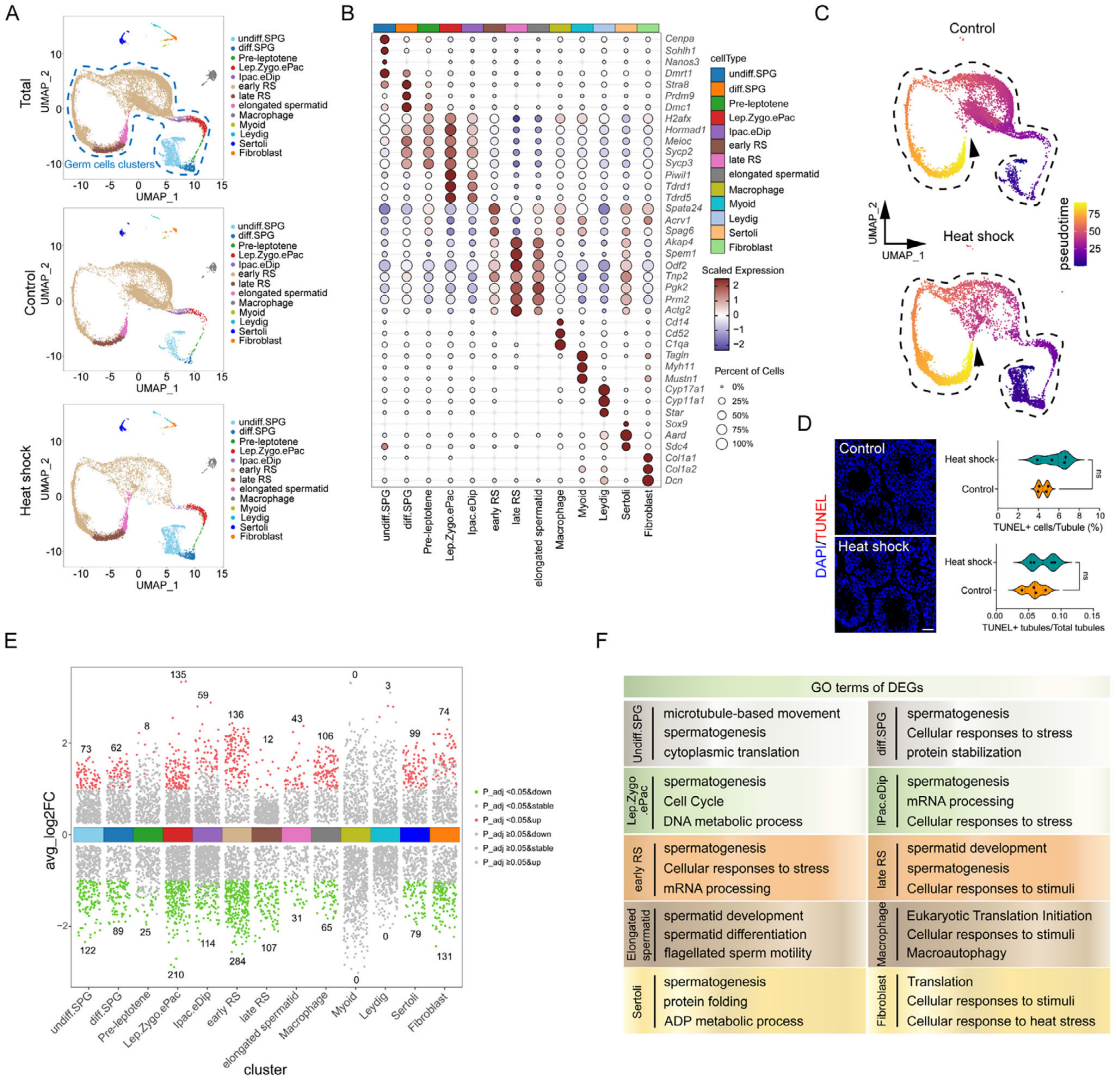
Constructing a single‐cell transcriptomic atlas of the mouse testis and analyzing its response to heat shock. (A) UMAP plot showing the 13 distinct cell clusters identified in the mouse testis, with annotations provided. The dataset was generated from integrated (total), control group, and heat‐shock group testicular single‐cell data, respectively. (B) A bubble plot displays the cell clusters validated by several cell type‐specifically expressed marker genes. The color gradient represents expression levels, and the bubble size indicates the percentage of cells expressing the gene. (C) Pseudotime trajectory of germ cell development, with the black dashed arrow showing the direction from spermatogonia to elongated spermatids. (D) TUNEL assay and quantitative analysis of apoptotic cells in testicular sections from control (*n *= 4) and heat stress (*n *= 4) mice. Statistical significance was assessed by unpaired two‐tailed Student's *t*‐tests. ns, not significant. Scale bar: 50 µm. (E) A single‐cell cluster volcano plot shows differentially expressed genes between the control and heat‐shock groups across different cell types. The *x*‐axis represents cell types, and the *y*‐axis indicates the log2FC of gene expression. Red dots denote upregulated genes, while green dots represent downregulated genes. The number of differentially expressed genes is labeled on the plot. (F) GO terms enriched by the differentially expressed genes within each cell type.

To overcome the confounding effects of cellular heterogeneity in testicular bulk RNA‐seq, we performed single‐cell RNA sequencing (scRNA‐seq) on both control and heat‐shocked testes, enabling a more precise assessment of the transcriptional impact of heat shock on germ cells. We analyzed a total of 13 001 cells from the control group and 10 721 cells from the heat‐shocked group. Unsupervised clustering analysis based on UMAP identified 13 transcriptionally distinct clusters, which were annotated as 8 germ cell and 5 somatic cell subpopulations using established marker genes (Figure 5A,B). We observed no substantial differences in cellular composition between the control and heat shock groups (Figure 5A), indicating that a 42°C heat shock for 30 min did not alter the overall testicular cell population. Furthermore, pseudotime trajectory analysis revealed a consistent germ cell developmental progression between the two groups (Figure 5C). This finding was further supported by TUNEL assays, which showed no significant increase in germ cell apoptosis following the heat shock (Figure 5D). Subsequent differential gene expression analysis across all 13 cell subpopulations revealed a relatively mild transcriptional response to heat shock (Figure 5E). The number of differentially expressed genes varied by cluster, ranging from 0 to 420 (Figure 5E). In germ cell subpopulations, DEGs were primarily associated with spermatogenesis and cellular responses to stress (Figure 5F). In contrast, macrophage and fibroblast clusters showed DEGs enriched for translation (Figure 5F). Furthermore, Sertoli cells exhibited DEGs linked to spermatogenesis (Figure 5F), suggesting a potential alteration in their supportive role for germ cells following heat shock. Collectively, our single‐cell transcriptomic data provide additional evidence supporting the finding that heat shock stress primarily impacts the testis at the level of translation, rather than inducing broad transcriptomic alterations.

### 2.8 BOULE Deficiency Impairs Stress Granule Disassembly and Compromises Germ Cell Survival Under Stress

**FIGURE 6:**
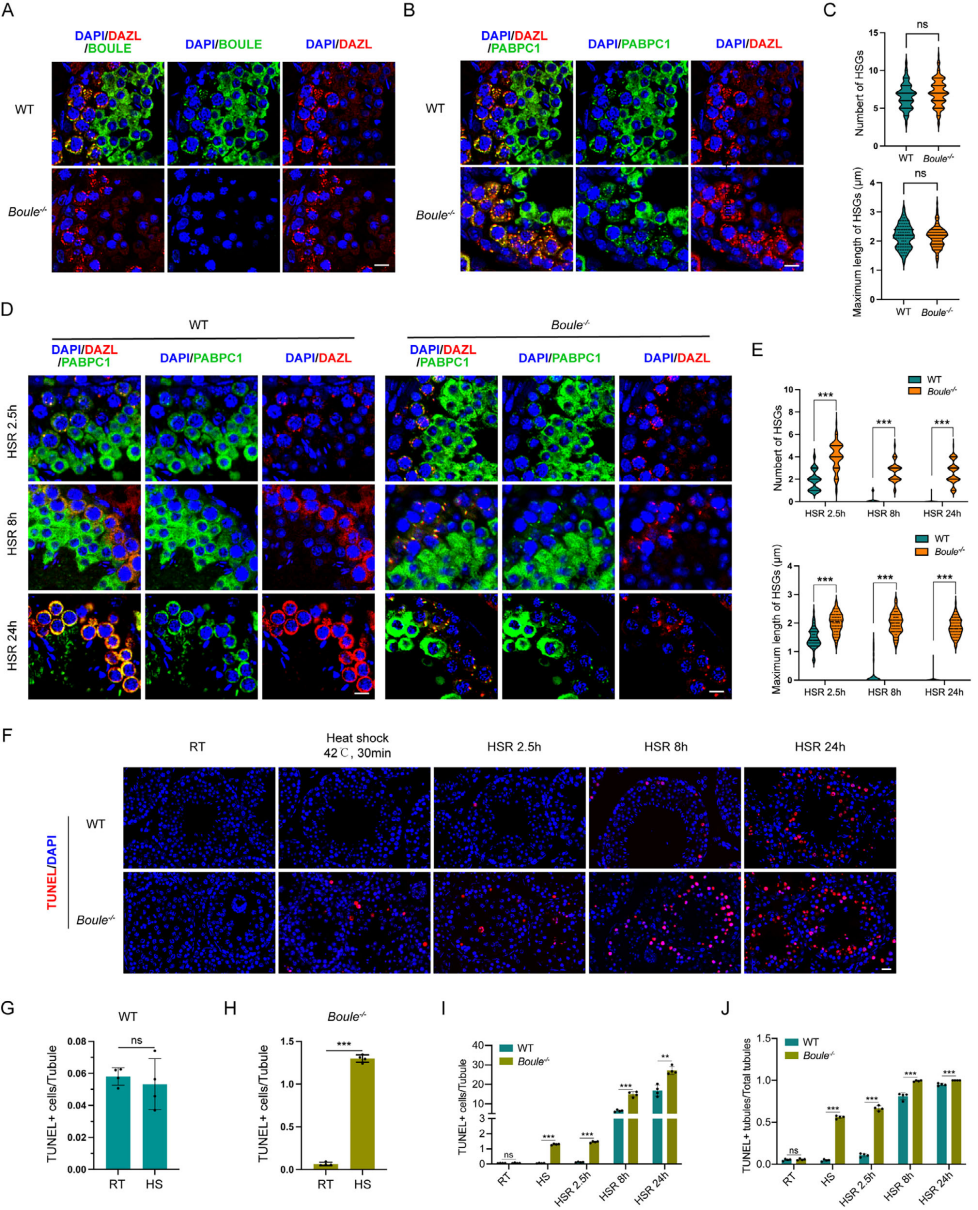
BOULE deficiency impairs stress granule disassembly and increases germ cell apoptosis during and after heat shock. (A) Immunofluorescence of testicular sections from heat‐shocked WT and *Boule* ^−/−^ mice, stained for DAZL (red) and BOULE protein (green). Nuclei are counterstained with DAPI. Scale bar: 10 µm. (B) Immunofluorescence of testicular sections from wild‐type (WT) and *Boule* ^−/−^ mice subjected to heat shock, stained for DAZL (red) and PABPC1 (green) to label heat stress granules. Nuclei are counterstained with DAPI (blue). Scale bar: 10 µm. (C) Quantification of the number and maximum diameter of heat shock‐induced stress granules in WT (*n* = 113 germ cells) and *Boule* ^−/−^ (*n* = 116 germ cells) testes. (D) Immunofluorescence of testicular sections from WT and *Boule^−/−^* mice at 2.5, 8, and 24 h of recovery at room temperature following heat shock (HSR), stained for DAZL (red) and PABPC1 (green). Nuclei are counterstained with DAPI. Scale bar: 10 µm. (E) Analysis of heat shock‐induced stress granules (number and maximum diameter) in germ cells from WT and *Boule^−/−^* testes during recovery. Data are derived from the number of germ cells indicated on the graph for each time point. (F) TUNEL assay on testicular sections from WT and *Boule^−/−^* mice at room temperature (RT), immediately after heat shock (HS), and after 2.5, 8, and 24 h of recovery. Nuclei (DAPI, blue) and TUNEL‐positive signals (red) are shown. Scale bar: 20 µm. (G–J) Analyses of apoptosis in testicular sections from WT and *Boule^−/−^* mice at room temperature (RT), immediately after heat shock (HS), and at the indicated recovery time points. (G) Quantification of apoptotic cells in WT testes. (H) Quantification of apoptotic cells in *Boule^−/−^* testes. (I) Number of apoptotic cells per seminiferous tubule (WT vs. *Boule^−/−^*). (J) Ratio of TUNEL‐positive seminiferous tubules (WT vs. *Boule^−/−^*). For all quantifications, data are from four mice per group and are presented as mean ± SD. Statistical significance was determined using unpaired two‐tailed Student's *t*‐tests (ns, not significant; **, *p* < 0.01; ***, *p* < 0.001).

Proteomic analysis of purified testicular stress granules revealed that they contain germ cell‐specific proteins such as DAZL, BOULE, DDX4, and PIWIL1, suggesting a unique molecular basis for these granules. While prior studies have established the critical role of DAZL in testicular HSG formation [41], the functions of other proteins in the dynamic assembly and disassembly of HSGs remain largely unexplored. To address this, we employed a *Boule^−/−^* mouse model to systematically investigate the impact of *Boule* deficiency on the assembly and disassembly kinetics of HSGs. Immunofluorescence analysis confirmed the complete absence of BOULE protein in *Boule^−/−^* testis sections (Figure 6A). Following heat shock at 42°C for 30 min, DAZL‐positive HSGs were still able to form in *Boule^−/−^* germ cells (Figure 6A). Co‐staining with another HSG marker, PABPC1, revealed prominent HSG formation and clear colocalization of DAZL and PABPC1 in both wild‐type and *Boule^−/−^* spermatocytes (Figure 6B). Quantitative analysis showed no significant difference in the number or size of HSGs between wild‐type and *Boule^−/−^* spermatocytes (Figure 6C), indicating that BOULE, although an HSG component, is dispensable for HSG assembly.

We next assessed the role of BOULE in HSG disassembly (Figure 6D). After 150 min of recovery at room temperature post‐heat shock, the number of HSGs in wild‐type spermatocytes decreased to approximately 2 per cell, whereas *Boule^−/−^* spermatocytes retained significantly more and larger HSGs (Figure 6E). Extending the recovery period to 8–24 h revealed a complete resolution of HSGs in wild‐type germ cells, with DAZL and PABPC1 displaying diffuse cytoplasmic distribution (Figure 6D). In contrast, *Boule^−/−^* germ cells still contained prominent HSGs after 8 h of recovery, and these granules persisted even after 24 h (Figure 6D). Quantification confirmed that both the number and size of HSGs in *Boule^−/−^* testes at these time points were significantly higher than in wild‐type controls (Figure 6E). These results demonstrate that BOULE deficiency specifically impairs the disassembly of heat shock‐induced HSGs.

To determine whether defective SG disassembly affects germ cell viability, we performed TUNEL assays on testicular sections from wild‐type and *Boule^−/−^* mice subjected to heat shock and recovery (Figure 6F). Without heat shock, *Boule^−/−^* testes showed no significant increase in TUNEL signal compared to wild‐type controls, indicating that BOULE loss alone does not induce apoptosis (Figure 6I,J). Notably, heat‐shocked *Boule^−/−^* mice exhibited a marked increase in apoptosis compared to both heat‐shocked wild‐type mice and untreated *Boule^−/−^* controls (Figure 6H–J), suggesting that *Boule*‐deficient germ cells have a reduced capacity to cope with external stress. Although apoptosis gradually increased in wild‐type testes during recovery (Figure 6G), *Boule^−/−^* testes demonstrated a more pronounced increase in apoptosis at all time points examined (Figure 6I,J). These findings further confirmed that BOULE deficiency compromises cell survival following stress.

### 2.9 BOULE Post‐Transcriptionally Promotes the Expression of Ubiquitination‐Related mRNAs in Germ Cells

**FIGURE 7:**
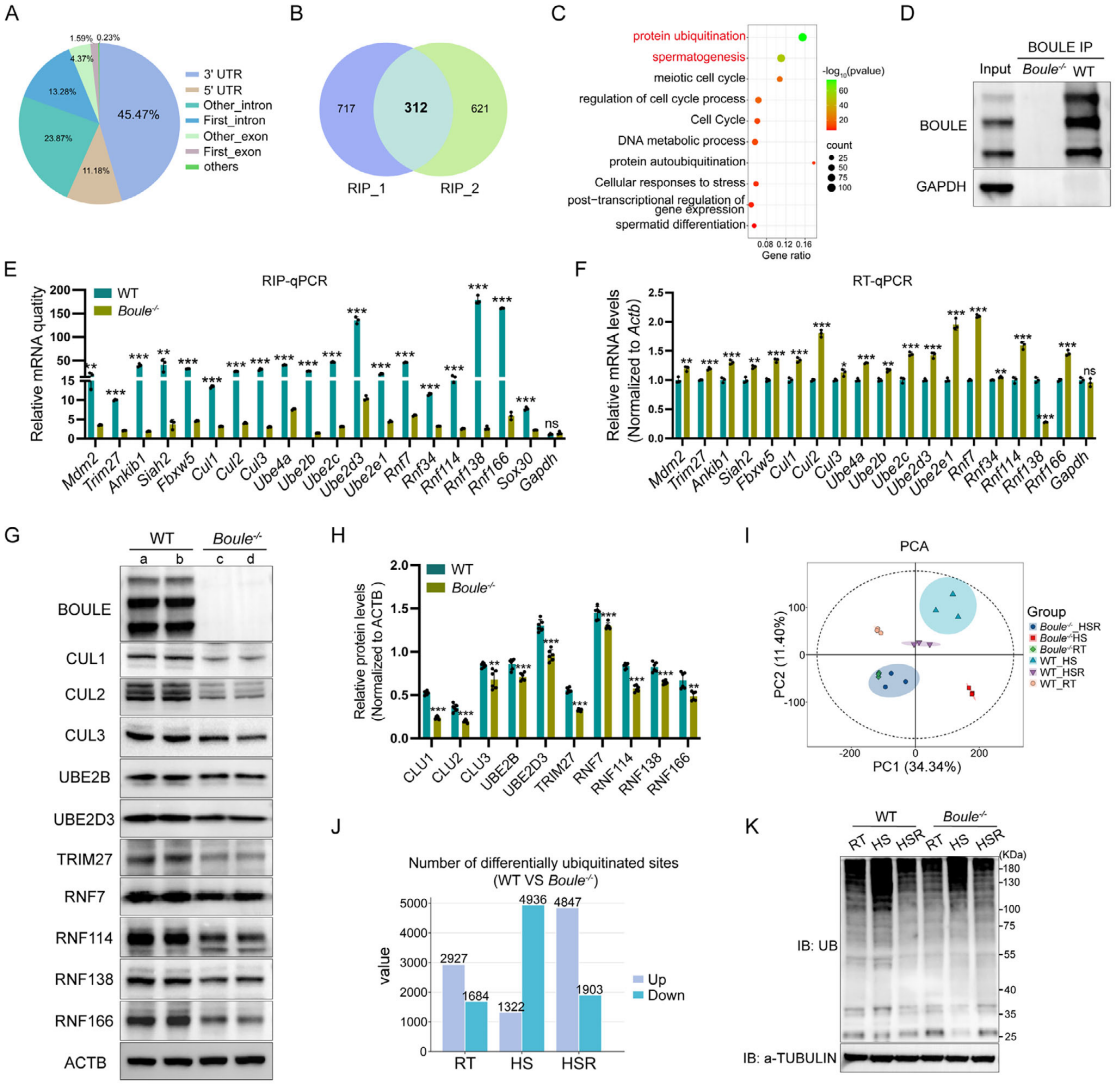
BOULE protein significantly enriches mRNAs of ubiquitination‐related genes, and its deficiency affects global ubiquitination modifications in the testes. (A) Pie chart showing the distribution of BOULE protein peaks across genomic functional elements. (B) Venn diagram illustrating the number of target genes identified by RIP‐seq in two biological replicates. (C) GO analysis of the 312 target genes of BOULE. (D) RNA immunoprecipitation of BOULE protein from wild‐type (WT) and *Boule^−/−^* testes, followed by western blot validation of BOULE enrichment. GAPDH served as a negative control. (E) RIP‐qPCR validation of the enrichment of ubiquitination‐related BOULE target genes in WT versus *Boule^−/−^* testes. *Sox30* was used as a positive control and *Gapdh* as a negative control. (F) RT‐qPCR analysis of ubiquitination‐related BOULE target mRNAs in WT and *Boule^−/−^* testes. *Gapdh* was used as an internal control. (G) Western blot detecting ubiquitination‐related proteins enriched by BOULE RNA immunoprecipitation in WT and *Boule^−/−^* testes. ACTB served as a loading control. Lanes a–d represent four individual mice. (H) Quantification of protein bands from (G) using ImageJ software. (I) Principal component analysis of ubiquitinated proteomics data from WT and *Boule^−/−^* testes under control, heat shock, and heat shock recovery (150 min) conditions. (J) Bar graph showing the number of differentially ubiquitinated sites in WT and *Boule^−/−^* testes under different treatment conditions. (K) Western blot analysis of total ubiquitination levels in WT and *Boule^−/−^* testes under indicated conditions. α‐Tubulin was used as a loading control. All experiments were analyzed by unpaired *t*‐test. Data are presented as mean ± SD. *p < 0.05, **p < 0.01, ***p < 0.001; ns, not significant.

To define the direct RNA interactome of BOULE and uncover the molecular basis for the impaired disassembly of heat shock granules in *Boule^−/−^* germ cells, we performed RNA immunoprecipitation sequencing (RIP‐seq) from murine testicular lysates. The results revealed that BOULE binding peaks were predominantly enriched in the 3' UTR regions of genomic functional elements (Figure 7A). Using a threshold of greater than twofold enrichment of RIP‐seq peaks in 3′ UTRs relative to input, a total of 312 BOULE target genes were identified across two biological replicates (Figure 7B; Data S3). Gene Ontology analysis of these target genes showed significant enrichment in terms such as protein ubiquitination, spermatogenesis, meiotic cell cycle, DNA metabolic process, and cellular response to stress (Figure 7C). We aimed to investigate whether BOULE is involved in regulating the expression of ubiquitin‐related proteins, based on the crucial role of ubiquitination in the disassembly of heat shock granules [14, 15, 16]. We then performed RIP‐qPCR to validate the RIP‐seq findings. Western blot analysis confirmed the successful immunoprecipitation of BOULE protein, with no specific signal detected in *Boule^−/−^* testis control (Figure 7D). Subsequently, RIP‐qPCR analysis demonstrated that BOULE specifically enriches multiple identified ubiquitin ligase mRNAs (Figure 7E). The specificity of the assay was further verified using *Sox30* (a known target) [47] and *Gapdh* (a non‐target) as positive and negative controls, respectively (Figure 7E). Intriguingly, transcript analysis in *Boule^−/−^* testes revealed an unexpected increase in mRNA levels for most validated targets, except for *Rnf138*, which showed a decrease (Figure 7F). In contrast, *Gapdh* mRNA levels remained unchanged (Figure 7F). We then examined the corresponding protein levels. Strikingly, despite the elevated mRNA levels, the abundance of several BOULE target proteins was markedly reduced in *Boule^−/−^* testes compared to wild‐type testes (Figure 7G,H). This inverse relationship between mRNA and protein levels suggests that BOULE acts as a post‐transcriptional regulator promoting the translation of ubiquitination‐related proteins.

### 2.10 BOULE Orchestrates Global Ubiquitination Dynamics in Testicular Germ Cells

To globally assess the impact of BOULE on the ubiquitination landscape in the testis, we performed ubiquitinated proteomic sequencing of wild‐type and *Boule^−/−^* testes under RT, HS, and HSR conditions (Data S4–S6). Principal component analysis of the ubiquitination profiles showed clear segregation among the three treatment groups in wild‐type mice (Figure 7I), demonstrating condition‐specific remodeling of the testicular ubiquitinome during stress granule dynamics. Notably, the ubiquitination profiles of *Boule^−/−^* testes formed a distinct cluster separate from all wild‐type groups across conditions (Figure 7I). Consistent with this observation, a quantitative comparison identified 4611, 6258, and 6750 differentially ubiquitinated sites between genotypes under RT, HS, and HSR conditions, respectively (Figure 7J), confirming that BOULE deficiency fundamentally alters the global ubiquitinome in testicular germ cells. We next examined global ubiquitination levels by western blot analysis of wild‐type and *Boule^−/−^* testicular lysates under RT, HS, and HSR conditions, with α‐tubulin serving as a loading control. In wild‐type testicular lysates, polyubiquitin conjugates increased transiently after heat shock and declined during recovery (Figure 7K). While *Boule^−/−^* lysates showed a parallel temporal pattern, the absolute abundance of ubiquitinated proteins was significantly reduced compared to wild‐type under both RT and HS conditions (Figure 7K). Therefore, BOULE may function as a key regulator of ubiquitination dynamics during heat shock in germ cells.

### 2.11 A Two‐Pronged Mechanism of BOULE Action: Promoting Expression of Core Components for Heat Shock Granule Disassembly and Licensing G3BP1 Ubiquitination

**FIGURE 8:**
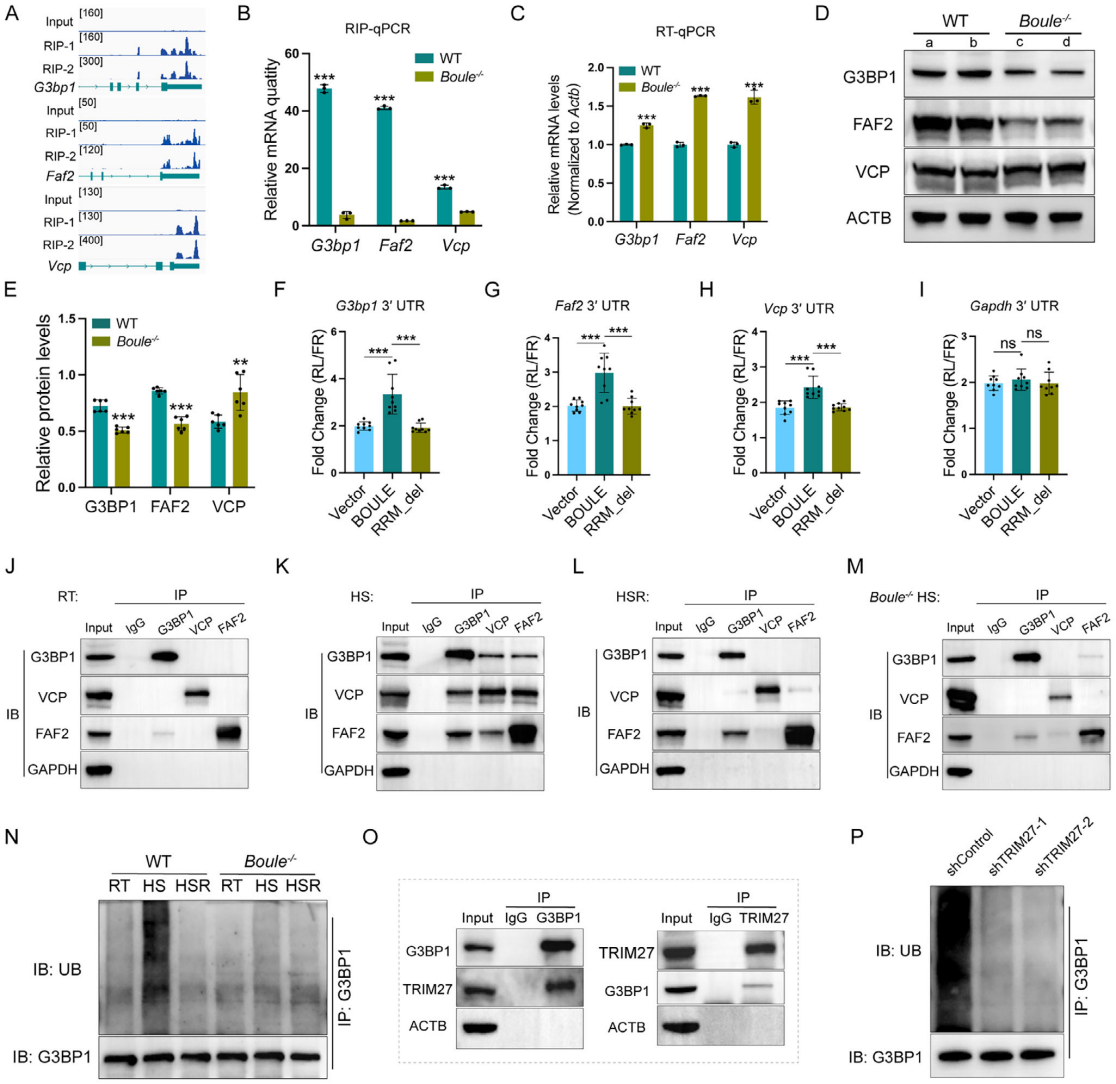
BOULE facilitates G3BP1 ubiquitination and G3BP1/FAF2/VCP complex formation in testes under heat shock. (A) Integrative Genomics Viewer (IGV) tracks showing BOULE protein binding peaks at the 3' UTRs of *G3bp1*, *Faf2*, and *Vcp* mRNAs. (B) RNA immunoprecipitation (RIP) of BOULE protein from wild‐type (WT) and *Boule^−/^* ^−^ testes, followed by RT‐qPCR analysis. *Actb* gene serves as the internal control. (C) RT‐qPCR analysis of *G3bp1*, *Faf2*, and *Vcp* transcript levels in WT and *Boule^−/^* ^−^ testes. (D) Western blot analysis of G3BP1, FAF2, and VCP protein expression in WT and *Boule^−/^* ^−^ testes. a‐d, representative of four individual mice. ACTB serves as the loading control. (E) Relative quantitative analysis of G3BP1, FAF2, and VCP protein levels from western blots in (D) using Image J software. (F–I) Dual luciferase reporter assays in HEK293T cells co‐transfected with the *G3bp1* 3' UTR (F), *Faf2* 3' UTR (G), *Vcp* 3' UTR (H), or *Gapdh* 3' UTR (I) constructs together with either an empty vector (Vector) plasmid, a full‐length BOULE expression plasmid (BOULE), or a plasmid expressing BOULE lacking the RRM domain (BOULE_RRM). (J–L) Co‐immunoprecipitation (Co‐IP) of G3BP1, FAF2, and VCP from WT testes under room temperature (RT) (J), heat shock (HS) (K), or HS recovery (L) conditions, followed by western blot analysis. GAPDH served as a negative control. (M) Co‐IP of G3BP1, FAF2, and VCP from *Boule^−/^* ^−^ testes after heat shock, followed by western blot analysis. GAPDH serves as a negative control. (N) G3BP1 Co‐IP from WT and *Boule^−/^* ^−^ testes under control, heat shock (HS), and HS recovery conditions, followed by western blot analysis for G3BP1 and Ubiquitin (UB). (O) Western blot showing the interaction between G3BP1 and TRIM27 in HEK293T cells. (P) Detection of G3BP1 ubiquitination in HEK293T cells following knockdown of TRIM27 using independent shRNAs (shTRIM27‐1 and shTRIM27‐2). The amount of immunoprecipitated G3BP1 was quantified and used as a loading control. Data are presented as mean ± SD. Statistical significance was determined by an unpaired two‐tailed *t*‐test. ***p < 0.001, **p < 0.01, ns, not significant.

Given the established role of the VCP/FAF2 complex in disassembling heat shock granules via extraction of ubiquitinated G3BP1 [14], we asked whether this classical disassembly pathway operates in testicular germ cells and is subject to regulation. Our RIP‐seq analysis revealed significant enrichment of BOULE binding peaks within the 3' UTRs of *G3bp1*, *Faf2*, and *Vcp* mRNAs (Figure 8A). These interactions were validated by testis‐specific RIP‐qPCR, confirming BOULE's direct association with these transcripts (Figure 8B). Despite a significant upregulation of *G3bp1*, *Faf2*, and *Vcp* mRNAs in *Boule^−/−^* testes (Figure 8C), we observed a discordant response at the protein level. Specifically, G3BP1 and FAF2 protein abundances were markedly reduced, whereas VCP protein levels were significantly elevated in *Boule^−/−^* testes (Figure 8D,E). The elevated VCP protein observed in *Boule^−/−^* testes is likely attributable to the >50% increase in its mRNA levels (Figure 8C), which may surpass a compensatory threshold in translation.

As expected, dual‐luciferase reporter assays demonstrated that BOULE directly enhanced the expression of reporters containing the 3' UTRs of *G3bp1* (1.7‐fold), *Faf2* (1.5‐fold), and *Vcp* (1.3‐fold) (Figure 8F–H). This activity was dependent on its RRM domain, whereas a control reporter with the *Gapdh* 3' UTRs was unaffected (Figure 8F–I), establishing BOULE as a specific, RNA‐binding‐dependent post‐transcriptional activator.

We next investigated the assembly of the RNA‐independent G3BP1/FAF2/VCP protein complex in testicular tissue under RT, HS, and HSR conditions via co‐immunoprecipitation (Co‐IP). While successful IP of each protein was confirmed under all conditions, a stable G3BP1/FAF2/VCP ternary complex was detected exclusively upon heat shock in wild‐type testes (Figure 8J–L). During the recovery phase, these interactions rapidly weakened and dissociated (Figure 8L). This assembly/disassembly cycle paralleled the dynamics of heat shock granule formation and clearance. Strikingly, this complex failed to assemble in heat‐shocked *Boule^−/−^* testes (Figure 8M). G3BP1 lost its ability to bind VCP, and its interaction with FAF2 was drastically weakened. Similarly, VCP failed to co‐precipitate G3BP1 and showed severely diminished binding to FAF2 (Figure 8M).

As the recruitment of the VCP/FAF2 complex to G3BP1 is dependent on G3BP1 ubiquitination [14], we analyzed this critical regulatory step. Our ubiquitinome data revealed a robust, transient increase in G3BP1 ubiquitination upon heat shock in wild‐type testes, which returned to baseline during recovery (Figure S9A). In contrast, *Boule^−/−^* testes exhibited only a minimal increase in G3BP1 ubiquitination under heat shock, representing a significant impairment compared to wild‐type (Figure S9A,B). This finding was independently confirmed by co‐immunoprecipitation of G3BP1 followed by ubiquitin immunoblotting. Using the level of immunoprecipitated G3BP1 as a loading control, the assay revealed a clear heat shock‐induced ubiquitination signal in wild‐type testes, which was absent in *Boule^−/−^* testes (Figure 8N).

### 2.12 TRIM27 Mediates BOULE‐Dependent G3BP1 Ubiquitination Under Heat Shock

Given that BOULE is required for heat‐shock‐induced G3BP1 ubiquitination (Figure 8N), we next sought to identify the responsible ubiquitination‐related proteins. We therefore focused on those genes bound by BOULE in our RIP‐seq data (Figure 7E). Co‐immunoprecipitation of G3BP1 from heat‐shocked wild‐type testicular lysates, followed by western blotting for candidate proteins, revealed that only TRIM27 co‐precipitated with G3BP1 among the tested proteins (Figure 8O; Figure S9C). The interaction was specific, as no signal was detected with control IgG, and the converse Co‐IP confirmed that TRIM27 pulled down G3BP1 (Figure 8O). To test whether TRIM27 directly promotes G3BP1 ubiquitination, we knocked down TRIM27 in HEK293T cells using two independent shRNAs (Figure S9D). Ubiquitination of immunoprecipitated G3BP1 was markedly reduced upon TRIM27 depletion (Figure 8P), with the amount of G3BP1 immunoprecipitate used as a loading control (Figure 8P). Together, these results indicate that TRIM27 acts as an E3 ligase to ubiquitinate G3BP1 upon heat shock. Given that Trim27 mRNA is bound and post‐transcriptionally regulated by the BOULE protein (Figure 7E–H), our results support a model in which BOULE licenses G3BP1 ubiquitination at least in part by promoting the expression of TRIM27.

**FIGURE 9:**
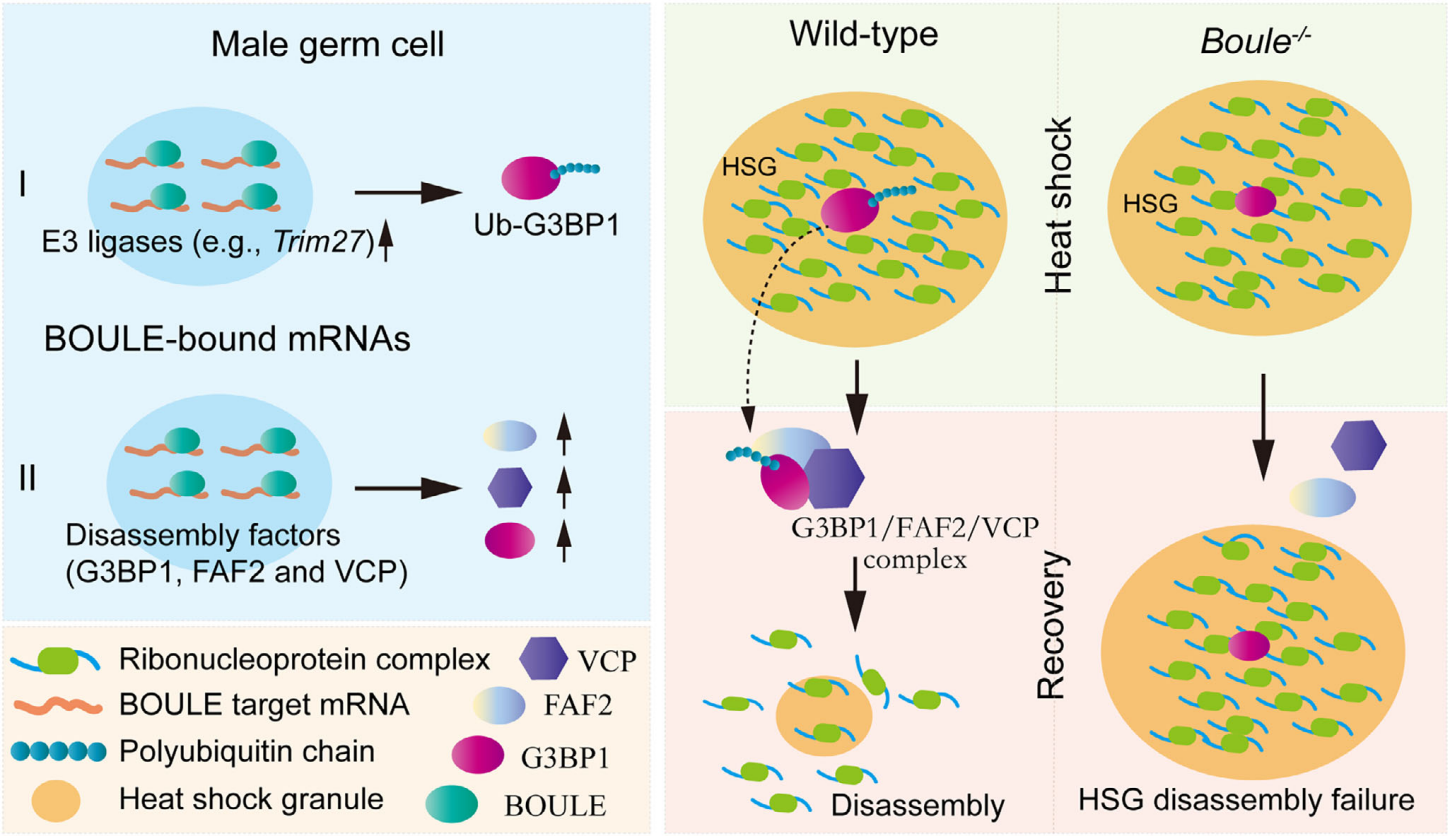
Schematic model illustrating the role of BOULE in heat shock granule disassembly in male germ cells.

In conclusion, our results define a two‐pronged mechanism for BOULE in orchestrating stress granule disassembly in germ cells. First, BOULE promotes the ubiquitination of G3BP1 by upregulating its target TRIM27, thereby generating the essential ubiquitin signal upon heat shock that licenses recognition by the VCP/FAF2 extractor complex. Second, BOULE post‐transcriptionally maintains the protein levels of G3BP1 and FAF2. Consequently, BOULE deficiency creates a combined defect that abrogates the assembly of a functional G3BP1/FAF2/VCP disassembly complex, ultimately leading to the failure of heat shock granule clearance (Figure 9).

## 3 Discussion

This study integrated multi‐omics analyses with a genetic knockout mouse model to systematically delineate the dynamic assembly and disassembly landscape of heat shock granules in the mammalian testis. We revealed that the germ cell‐specific RNA‐binding protein BOULE functions as a critical orchestrator for the timely clearance of HSGs and subsequent cell survival. Rather than simply contributing to disassembly, BOULE controls this process through a sophisticated two‐pronged mechanism: first, by binding and enhancing the translation of mRNAs encoding core disassembly factors such as *G3bp1* and *Faf2*, thus maintaining a functional pool of clearance machinery components; second, by enabling heat shock‐induced ubiquitination of G3BP1, a prerequisite for recruiting the VCP/FAF2 complex. This finding expands the functional repertoire of BOULE from a conventional executor of developmental programs to a guardian of cellular homeostasis under environmental stress, establishing a new paradigm for understanding the molecular basis of stress‐induced male infertility.

BOULE, the most evolutionarily conserved member of the DAZ family, is specifically expressed in male germ cells and is indispensable for spermatogenesis [47, 48, 49, 50]. Intriguingly, BOULE protein expression initiates in spermatocytes, yet *Boule^−/−^* mice exhibit normal meiosis progression but suffer a spermatogenic arrest at the round spermatid stage [50]. The functional role of BOULE protein during the spermatocyte stage has remained elusive. Pachytene spermatocytes, particularly early stages, are critical for homologous recombination and DNA repair. We propose that efficient HSG formation and their subsequent BOULE‐dependent clearance represent an adaptive strategy by which spermatocytes cope with heat shock, potentially protecting genomic integrity during meiosis. A key concern is whether the SG disassembly defects observed in *Boule^−/−^* germ cells are indirect consequences of altered testicular cell composition. To address this, we performed scRNA‐seq on wild‐type and *Boule^−/−^* testes. Apart from the expected absence of elongated spermatids, we found no significant changes in overall cellular composition across any other germ cell or somatic cell type (Figure S10A). Critically, within each matched cell population, we observed no widespread transcriptomic changes between genotypes, identifying fewer than 30 differentially expressed genes per cluster (|fold change| > 2, *p* < 0.05) (Figure S10B). These results rule out indirect effects from cell composition changes or secondary transcriptional alterations as the primary cause of the observed HSG disassembly defects. Thus, BOULE likely acts directly in germ cells to promote HSG clearance and facilitate meiotic progression under heat shock. Moreover, a recent study showed that under heat shock, *circBoule* promotes the ubiquitination and degradation of heat shock proteins, indicating that fine‐tuning of the heat shock response is crucial for normal sperm development [51]. Therefore, both the protein and the circular RNA produced from the *Boule* locus appear to function in protecting testicular germ cells from heat shock.

Through purification of HSG cores, we revealed that testicular HSGs possess a distinct proteome enriched with germ cell‐specific RNA‐binding proteins, including DAZL, BOULE, DDX4, PIWIL1, and LIN28A, which play essential roles in spermatogenesis [50, 52, 53, 54, 55]. These components not only define a unique molecular identity for germ cell HSGs but also suggest that their roles may extend beyond conventional translational repression. This notion was strongly supported by our Ribo‐seq data. In contrast to the widespread translational suppression typically observed in stressed somatic cells, genes associated with spermatogenesis, flagellated sperm motility, and spermatid development were either translationally maintained or even upregulated upon heat shock in germ cells (Figure 4D). This pattern indicates that male germ cells employ a selective stabilization strategy under environmental stress, whereby core differentiation programs are prioritized within a globally repressed translational landscape, rather than undergoing a blanket translational shutdown. These observations align with emerging evidence in somatic systems [9, 24, 25], which challenges the traditional paradigm of HSGs as static sites of mRNA storage and silencing, and instead positions them as dynamic hubs for post‐transcriptional mRNA management.

This study provides the first systematic characterization of heat shock granule dynamics in mammalian germ cells. It reveals their unique properties, which include stage‐specific formation patterns, a specialized core proteome, and a germ cell‐specific disassembly pathway, collectively distinguishing them from somatic HSGs. This significantly expands our understanding of the diversity of cellular stress responses. More importantly, we redefine the function of the well‐known germline regulator BOULE as a switch for restoring cellular homeostasis under environmental pressure, offering a novel molecular explanation for its essential role in spermatogenesis. We acknowledge that the relative contribution of reduced G3BP1/FAF2 abundance versus defective ubiquitination to the impaired disassembly complex in *Boule^−/−^* germ cells remains unclear. Nonetheless, our data establish that BOULE operates at multiple steps of heat shock granule disassembly. First, BOULE post‐transcriptionally maintains G3BP1, FAF2, and VCP expression by binding directly to their 3′ UTRs and promoting translation (Figure 8A–H). Second, BOULE enables heat‐shock‐induced G3BP1 ubiquitination, at least in part by regulating the E3 ligase TRIM27 (Figure 8P). Consequently, BOULE deficiency compromises both the protein abundance of these disassembly factors and the ubiquitination signal that recruits VCP/FAF2 to G3BP1. Whether the reduced protein levels of G3BP1 and FAF2 alone directly cause the assembly defect remains an open question, but our results place BOULE as a multi‐level coordinator of expression, modification and complex assembly. This multilayered regulatory mechanism highlights the robustness of germ cell stress responses and explains why BOULE is indispensable for spermatogenic homeostasis under environmental stress.

Our study also has several limitations. First, the 42°C for 30 min testis heat shock paradigm models an acute hyperthermia episode. Whether the BOULE‐mediated regulation of HSG dynamics and ubiquitination is similarly engaged during chronic, lower‐grade heat exposure (e.g., lifestyle‐related scrotal hyperthermia or varicocele) or in human clinical contexts remains unknown and warrants future investigation. The functional mode and significance of this pathway under chronic mild heat exposure or other stress inducers also remain to be elucidated. Second, BOULE regulates a broad network of target mRNAs. While we elucidated its regulation of ubiquitin‐system components, how it coordinates the translation of other target genes (e.g., those involved in spermatogenesis and meiosis) and synergizes with the ubiquitin pathway to ensure overall cellular adaptation requires more systematic investigation. Furthermore, this work was conducted primarily in a mouse model. The conservation and clinical relevance of this pathway in human germ cells warrant further validation using human samples. Future studies addressing these aspects will deepen our understanding of the environmental adaptation mechanisms in germ cells and provide new insights and potential targets for the precise diagnosis and treatment of male infertility.

## 4 Materials and Methods

### 4.1 Animals and Heat Shock Treatment

All animal procedures were approved by the Animal Care and Use Committee (ACUC) of Nanjing Medical University (Ethics Approval Number: IACUC‐2112011). Eight‐week‐old male mice were subjected to heat shock under anesthesia by immersing the lower one‐third of the body in a water bath at 42°C for 30 min. Following heat shock, mice were allowed to recover at room temperature for specified durations: 30, 60, 90, 120, and 150 min, as well as 8 and 24 h. Immediately after either heat shock or the designated recovery period, mice were euthanized, and testicular tissues were collected for subsequent histological and molecular biological analyses.

The C57BL/6 inbred *Boule^−/−^* mouse line, previously described [56], was used in this study. *Boule^+/−^* mice were kindly provided by Professor Eugene Yujun Xu from the University of Chicago. Homozygous *Boule^−/−^* offspring were generated by crossing heterozygous breeders. Genotyping was performed via PCR amplification of genomic DNA extracted from mouse toe biopsies.

### 4.2 Isolation of Heat‐Shock Granule Core Components

The procedure for isolation of heat‐shock granule cores was adapted from previously established methods [43, 44, 45]. Briefly, dissected mouse testes were immediately homogenized in 1 mL of SG lysis buffer (50 mm Tris‐HCl, pH 7.4, 100 mm KOAc, 2 mm MgOAc, 0.5 mm DTT, 50 µg/mL heparin, 0.5% NP‐40) supplemented with 1× protease inhibitor cocktail (Roche) and 250 IU/mL RNase Inhibitor (Promega). Homogenization was performed using gentle sonication, followed by rotation at 4°C for 30 min to complete cell lysis. The lysate was centrifuged at 1000 × *g *for 5 min at 4°C. Subsequently, 10% of the resulting supernatant was transferred to a new tube for total RNA or protein extraction, while the remaining 90% was used for SG RNA or SG protein isolation.

For HSG core enrichment, 900 µL of the supernatant was subjected to sequential centrifugation. The sample was first centrifuged at 20 000 × *g* for 20 min at 4°C. The pellet was resuspended in 1 mL of SG lysis buffer and centrifuged again under the same conditions. The resulting pellet was resuspended in 500 µL of SG lysis buffer, yielding the HSG core‐enriched fraction. This HSG core‐enriched fraction was incubated with 50 µL of Protein G Dynabeads (Invitrogen), which had been pre‐washed with SG lysis buffer, for 30 min at 4°C with rotation. After magnetic separation, the pretreatment supernatant was incubated with specific antibodies (either anti‐BOULE or anti‐PABPC1) for 4 h at 4°C with gentle rotation. Following antibody incubation, the immune complexes were then pelleted by centrifugation at 18 000 × *g* for 20 min at 4°C. The pellet was resuspended in 500 µL of SG lysis buffer and incubated with 150 µL of pre‐washed Protein G Dynabeads overnight at 4°C with continuous rotation.

On the following day, the bead‐bound complexes were washed three times for 5 min each at 4°C with Wash Buffer 1 (20 mm Tris‐HCl, pH 8, 200 mm NaCl, 1 U/µL RNase Inhibitor), followed by three additional 5‐min washes at 4°C with Wash Buffer 2 (20 mm Tris‐HCl, pH 8, 500 mm NaCl, 1 U/µL RNase Inhibitor). Samples intended for data‐independent acquisition (DIA) mass spectrometry analysis were collected at this stage. For samples designated for transcriptomic analysis, the beads were subjected to three additional 2‐min washes at 4°C using Wash Buffer 3 (2 m urea, 1 U/mL RNase Inhibitor). RNA was then eluted by incubating the beads in 200 µL of Proteinase K solution (100 µg/mL Proteinase K in 1× TE buffer containing 2 m urea and 1 U/mL RNase Inhibitor) at 37°C for 15 min with shaking. Subsequently, 1 mL of TRIzol was added and incubated at 4°C with rotation for 5 min to elute RNA. Following magnetic separation of the beads, HSG RNA was extracted from the TRIzol‐containing supernatant using a commercial kit for transcriptome sequencing analysis.

### 4.3 Ribosome Profiling

Ribosome profiling was performed based on established protocols with a few modifications [57]. Testicular tissues from control, heat shock (HS), and heat shock recovery (HSR) groups were immediately snap‐frozen in liquid nitrogen. Samples were ground to powder in liquid nitrogen using a mortar and pestle, then homogenized in 400 µL of lysis buffer. The lysate was passed through a 26‐G needle ten times and clarified by centrifugation at 20 000 × *g* for 10 min at 4°C. To isolate ribosome footprints (RFs), the supernatant was treated with 10 µL of RNase I (NEB) and 6 µL of DNase I (NEB) at room temperature for 45 min with gentle agitation. Nuclease digestion was terminated by adding 10 µL of SUPERase‐In RNase Inhibitor (Ambion). The digest was purified using illustra MicroSpin S‐400 HR columns (GE Healthcare) and RFs longer than 17 nt were selected with an RNA Clean & Concentrator‐25 kit (Zymo Research). Ribosomal RNA was depleted as previously described (Morlan et al., 2012) by hybridization with antisense DNA probes complementary to rRNA sequences, followed by RNase H and DNase I (NEB) treatment, and final purification using magnetic beads (Vazyme).

Ribo‐seq libraries were constructed from the isolated RFs using the NEBNext Multiple Small RNA Library Prep Set for Illumina (NEB, catalog no. E7300S/L). Adapters were ligated to both ends of the RFs, followed by reverse transcription and PCR amplification. cDNA libraries with insert sizes of 140–160 bp were size‐selected and sequenced on an Illumina NovaSeq X Plus platform at Gene Denovo Biotechnology Co., Ltd. (Guangzhou, China).

### 4.4 Testicular Single‐Cell Suspension Preparation and 10× Genomics Sequencing

Testicular tissues from 8‐week‐old mice of different experimental groups were minced into small fragments. Tissue fragments were digested in Hanks' Balanced Salt Solution (HBSS, Gibco) containing collagenase for 15 min at 37°C. The digestion mixture was centrifuged at 600 × *g* for 1 min. The resulting pellet was resuspended in pre‐warmed 0.25% trypsin solution (Gibco) supplemented with 20% DNase I (Sigma) and pipetted repeatedly for 2 min using a 10 mL serological pipette, followed by incubation at 37°C for 5 min. The tissue mixture was pipetted for an additional 2 min and further incubated at 37°C for 3 min. Enzymatic digestion was terminated by adding fetal bovine serum (FBS, Sigma). Cells were washed twice in PBS, each wash performed by centrifugation at 600 × *g* for 5 min. The resulting cell suspension was filtered through a 40 µm cell strainer to obtain a single‐cell suspension.

Cell quality was assessed by counting and determining viability using Trypan Blue exclusion. The cell suspension was loaded onto a 10× Genomics GemCode Single‐cell instrument to generate single‐cell gel beads‐in‐emulsions. Gel beads were dissolved to release barcode sequences, followed by reverse transcription within each droplet to generate barcoded full‐length cDNA. Sequencing libraries were constructed from the amplified cDNA according to the manufacturer's protocol. Briefly, cDNA was fragmented to an average size of 200–300 bp. Sequencing adapters were added using a standard next‐generation sequencing library preparation workflow, followed by PCR amplification. Libraries were sequenced on an Illumina NovaSeq X Plus platform using paired‐end sequencing. Read 1 contained the 16‐bp 10× Barcode and the 10‐bp unique molecular identifier (UMI) for cell demultiplexing and digital gene‐expression quantification. Read 2 contained the cDNA‐derived insert for alignment to the reference genome to determine gene identity.

### 4.5 Histological Processing, Immunofluorescence Staining, and Heat Shock Granule Analysis

Testes harvested from mice of different experimental groups were fixed by immersion in Hartman's fixative (Sigma) under gentle agitation for 24 h. Each testis was then bisected, and fixation was continued under continuous agitation for an additional 24 h. After fixation and three 15‐min washes in 70% ethanol, the tissues were dehydrated through a graded ethanol series, cleared in xylene, and embedded in paraffin blocks. Finally, sections of 5 µm thickness were obtained using a rotary microtome.

For immunofluorescence, paraffin sections were first deparaffinized in xylene and rehydrated through a graded ethanol series. To quench endogenous peroxidase activity, sections were treated with 3% hydrogen peroxide (prepared in methanol) for 10 min. Antigen retrieval was performed by heating the sections in sodium citrate buffer. Nonspecific binding sites were blocked by incubation with 5% bovine serum albumin (BSA, Sigma) at room temperature for 2 h. The sections were subsequently incubated overnight at 4°C with primary antibodies diluted in blocking buffer. The primary antibodies used were as follows: mouse anti‐DAZL (AbD Serotec, MCA2336, 1:50), rabbit anti‐PABPC1 (Abcam, ab21060, 1:100), rabbit anti‐G3BP1 (Proteintech, 13057‐2‐AP, 1:100), and rabbit anti‐BOULE (a gift from Dr. Eugene Yujun Xu, University of Chicago, 1:500). The following day, sections were washed with 1 × PBS (Gibco, USA) and incubated with appropriate fluorophore‐conjugated secondary antibodies (Jackson ImmunoResearch, USA) for 1 h at room temperature protected from light. Following another wash cycle with PBS, cell nuclei were counterstained with DAPI (MCE, USA), and the sections were finally mounted with an anti‐fade mounting medium (MCE, USA).

Whole‐slide scanning was performed using a 3DHistech (Hungary) panoramic slide scanner. Subsequent quantitative analysis of stress granules (SGs) was performed using the CaseViewer software (3DHistech, Hungary). Seminiferous tubules at specific stages of the spermatogenic cycle were first identified based on their well‐defined cellular composition and characteristic morphology, as previously described [46]. Germ cell types within these staged tubules were then discerned using established cytological criteria. Within the defined tubule stages, HSG‐like cytoplasmic foci immunopositive for specific markers (e.g., DAZL, PABPC1) were identified. The number of these distinct HSGs per tubule cross‐section was manually counted. To assess HSG size, the maximum diameter (the longest axis) of individual, well‐defined granules was measured using the integrated measurement tool in the CaseViewer software. Measurements were performed at high magnification to ensure accuracy.

### 4.6 TUNEL Assay

The TUNEL assay was performed to detect apoptotic cells in testicular tissue, as previously described [58]. Briefly, paraffin‐embedded testicular sections were deparaffinized in xylene and rehydrated through a graded ethanol series. After three 5‐min washes in 1 × DPBS (Gibco), the sections were treated with proteinase K (20 µg/mL; Beyotime) for 30 min at room temperature, followed by another three 5‐min washes with 1 × DPBS. Apoptotic cells were labeled using the TUNEL BrightGreen Apoptosis Detection Kit (Vazyme) according to the manufacturer's instructions. Nuclei were counterstained with DAPI (Sigma). Sections were imaged under a confocal laser scanning microscope (Zeiss LSM 800). TUNEL‐positive cells were quantified for statistical analysis.

### 4.7 Co‐Immunoprecipitation (Co‐IP) Assay

Testicular tissues were homogenized in 1 mL of ice‐cold lysis buffer (50 mm Tris‐HCl pH 7.5, 100 mm KCl, 12 mm MgCl_2_, 1% NP‐40, 1 mm DTT, 1× protease inhibitor cocktail, 200 U/mL RNase inhibitor) using a mechanical homogenizer. The homogenates were incubated with rotation for 30 min at 4°C to complete lysis, followed by centrifugation at 20 000 × *g* for 20 min at 4°C. The supernatant was collected, and 5% of the volume was saved as the input control. The remaining supernatant was incubated with either the target‐specific antibody or control normal IgG (Beyotime) for 6 h at 4°C under gentle rotation. Protein G Dynabeads (Invitrogen) were equilibrated by washing three times with the wash buffer (50 mm Tris‐HCl pH 7.5, 100 mm KCl, 12 mm MgCl2, 1% NP‐40, 1× protease inhibitor cocktail). The antibody‐lysate mixture was then added to the pre‐equilibrated Dynabeads and incubated overnight at 4°C with rotation. The next day, the Dynabeads were collected using a magnetic stand (Invitrogen), and the supernatant was discarded. The Dynabeads were washed twice with ice‐cold wash buffer, followed by three washes with a high‐salt wash buffer (50 mm Tris‐HCl pH 7.5, 300 mm KCl, 12 mm MgCl2, 1% NP‐40), and finally once with the wash buffer. To disrupt potential RNA‐mediated protein interactions, the Dynabeads were resuspended in RNase A‐containing wash buffer and incubated at 37°C for 15 min. After treatment, the beads were washed once with ice‐cold wash buffer. Bound proteins were eluted by resuspending the Dynabeads in 1 × SDS‐PAGE loading buffer and heating at 95°C for 10 min. Both the input samples and the Co‐IP eluates were analyzed by Western blotting to detect co‐precipitated proteins. In the Co‐IP assay, secondary antibodies specific to either the light chain or heavy chain were used (Abmart) to minimize detection interference from antibody chains.

### 4.8 Western Blot Analysis

Protein samples were prepared using RIPA lysis buffer (50 mm Tris‐HCl, pH 7.4, 150 mm NaCl, 0.5% sodium deoxycholate, 1% NP‐40) supplemented with a protease inhibitor cocktail (Roche, 11697498001). The detailed procedure was performed as previously described [58]. The following primary antibodies were used for immunoblotting: mouse anti‐DAZL (AbD Serotec, MCA2336, 1:1000), Rabbit anti‐α‐Tubulin (1:1000; sc‐8035, Santa Cruz Biotechnology), Mouse anti‐ACTB (1:5000, 66009‐1‐Ig, Proteintech), Mouse anti‐GAPDH (1:5000, 60004‐1‐Ig, Proteintech), Rabbit anti‐G3BP1 (1:1000, 13057‐2‐AP, Proteintech), Rabbit anti‐G3BP1 (1:1000, R24375, Zenbio), Rabbit anti‐PABPC1 (1:1000, ab21060, Abcam), Rabbit anti‐TRIM27 (1:1000, 12205‐1‐AP, Proteintech), Rabbit anti‐CUL1 (1:1000, R24008, Zenbio), Rabbit anti‐CUL2 (1:1000, R383096, Zenbio), Rabbit anti‐CUL3 (1:1000, R382186, Zenbio), Rabbit anti‐UBE2B (1:1000, R30081, Zenbio), Rabbit anti‐UBE2D3 (1:1000, R26019, Zenbio), Rabbit anti‐RNF7 (1:1000, R30185, Zenbio), Rabbit anti‐RNF114 (1:1000, 14338‐1‐AP, Proteintech), Rabbit anti‐RNF7138 (1:1000, 17039‐1‐AP, Proteintech), Rabbit anti‐RNF166 (1:1000, 671741, Zenbio), and Rabbit anti‐UB (1:1000, 10201‐2‐AP, Proteintech). After washing, the membranes were incubated with horseradish peroxidase (HRP)‐conjugated secondary antibodies at room temperature for 1 h. Protein signals were detected using an enhanced chemiluminescence (ECL) substrate (Vazyme) and visualized with a Tanon imaging system (Tanon). ACTB, α‐Tubulin, and GAPDH were used as loading controls to normalize protein expression levels.

### 4.9 Quantitative Real‐Time PCR (RT‐qPCR)

Total RNA was isolated from testis samples using Trizol Reagent (Ambion) according to the manufacturer's instructions. Complementary DNA (cDNA) was synthesized from 500 ng of total RNA using the PrimeScript RT Master Mix (TaKaRa). Real‐time quantitative PCR was performed on a StepOnePlus Detection System (Applied Biosystems) using the ChamQ SYBR Color qPCR Master Mix (Vazyme) with a two‐step PCR amplification protocol. All primer sequences used in this study are listed in Data S7.

### 4.10 Dual‐Luciferase Reporter Assay

Full‐length mouse *Boule* coding sequence and a mutant lacking the RNA recognition motif (RRM) domain (RRM_del) were cloned into the mammalian expression vector pCMV6 to generate effector plasmids. The 3′ untranslated regions (3′ UTRs) of putative target genes (*G3bp1*, *Faf2*, and *Vcp*) were amplified by PCR from mouse genomic DNA and individually inserted downstream of the Renilla luciferase gene in the psiCHECK‐2 reporter vector (Promega). The empty psiCHECK‐2 vector served as a negative control. NIH/3T3 cells were seeded into 24‐well plates at a density of 2 × 105 cells per well and cultured for 24 h. Cells were co‐transfected with 150 ng of the psiCHECK‐2 reporter plasmid and 450 ng of an effector plasmid (pCMV6‐BOULE, pCMV6‐RRM_del, or pCMV6‐vector) using FuGENE HD transfection reagent (Promega) according to the manufacturer's instructions. At 48 h post‐transfection, firefly and Renilla luciferase activities were measured sequentially using a Dual‐Luciferase Reporter Assay System (Vazyme). All experiments were performed in triplicate and repeated at least three times independently.

### 4.11 RNA Immunoprecipitation Sequencing (RIP‐seq)

Testes were collected from mice and homogenized in ice‐cold lysis buffer (50 mm Tris‐HCl, pH 7.5, 100 mm KCl, 12 mm MgCl_2_, 1% NP‐40, 1 mm DTT, 1× protease inhibitor cocktail, and 200 U/mL RNase inhibitor) using a Dounce homogenizer to isolate seminiferous tubules. The tubules were subjected to UV crosslinking at 254 nm. Subsequently, cells were disrupted by sonication, and the lysate was incubated under gentle rotation at 4°C for 30 min. Following centrifugation at 20 000 × *g* for 20 min at 4°C, 10% of the supernatant was saved as input control. The remaining lysate was incubated with either an anti‐BOULE antibody or a control rabbit IgG overnight. RNA immunoprecipitation was performed according to the manufacturer's instructions using the RNA Immunoprecipitation Kit (Engibody). RNA extracted from input and anti‐BOULE immunoprecipitated samples was used for strand‐specific library construction with the TruSeq Stranded mRNA Library Prep Kit (Illumina). Library quality and fragment size distribution were assessed using a Qseq100 Bio‐Fragment Analyzer (Bioptic). Sequencing was carried out on an Illumina NovaSeq 6000 platform, generating 2 × 150 bp paired‐end reads.

### 4.12 Ubiquitin Proteomics

Ubiquitin proteomic analysis was performed by Westlake Omics Inc (China). Briefly, tissue samples were ground in liquid nitrogen and lysed in a buffer containing 4% SDS, 100 mm DTT, and 150 mm Tris‐HCl (pH 8). The lysates were sonicated and centrifuged, and the supernatants were collected for protein quantification using a BCA assay (Thermo Scientific). Protein digestion was conducted using the filter‐aided sample preparation (FASP) method. After digestion with trypsin (Sigma‐Aldrich, Germany), peptides were collected by centrifugation, acidified with formic acid, dried, and desalted using C18 Stage Tips (Thermo Scientific). Ubiquitin‐modified peptides were enriched using a PTMScan Ubiquitin Remnant Motif (K‐ε‐GG) Kit (CST). Peptides were incubated with K‐ε‐GG‐specific antibody beads, washed, and eluted with 0.15% trifluoroacetic acid (TFA), followed by desalting with C18 Stage Tips. Liquid chromatography‐tandem mass spectrometry (LC‐MS/MS) was performed using a Vanquish Neo UHPLC system (Thermo Scientific) coupled to an Orbitrap Astral mass spectrometer (Thermo Scientific). Peptides were separated on a 50‐cm µPAC Neo column (Thermo Scientific) with an 8‐min acetonitrile gradient. Data were acquired in DIA mode.

### 4.13 G3BP1 Ubiquitination Assay

To examine G3BP1 ubiquitination in testicular tissue, wild‐type and *Boule^−/−^* mouse testes were collected under control, heat shock, and heat shock recovery conditions. Tissues were homogenized in RIPA lysis buffer (50 mm Tris‐HCl pH 7.4, 150 mm NaCl, 0.5% sodium deoxycholate, 1% NP‐40) supplemented with 1× protease inhibitor cocktail and 10 mm N‐ethylmaleimide (NEM, Sigma) to inhibit deubiquitination. G3BP1 was immunoprecipitated using anti‐G3BP1 antibody or control IgG, followed by western blotting with anti‐ubiquitin antibody. The amount of immunoprecipitated G3BP1 served as a loading control.

For TRIM27 knockdown, HEK293T cells were transiently transfected with two independent shRNAs targeting TRIM27 (shTRIM27‐1 and shTRIM27‐2) or control shRNA as previously described [59]. After 48 h, TRIM27 knockdown efficiency was confirmed by western blotting. Cells were then lysed in 8 m urea lysis buffer (50 mm Tris‐HCl pH 7.5, 150 mm NaCl, 8 m urea, 10 mm NEM, 1× protease inhibitor cocktail), and endogenous G3BP1 was immunoprecipitated using anti‐G3BP1 antibody. Ubiquitination of G3BP1 was detected by anti‐ubiquitin western blotting, with the amount of immunoprecipitated G3BP1 as a loading control.

### 4.14 Statistics and Reproducibility

Statistical analysis was performed using GraphPad Prism 9 (GraphPad Software, USA). Quantitative data are presented as the mean ± standard deviation (SD). For comparisons involving more than two groups, one‐way analysis of variance (ANOVA) followed by Tukey's post hoc test was applied to adjust for multiple comparisons. Pairwise comparisons between two specific groups were performed using unpaired two‐tailed Student's *t*‐tests. A *p*‐value < 0.05 was considered statistically significant. All experimental procedures, including histology, immunofluorescence, western blot, immunoprecipitation, TUNEL assay, and RT‐qPCR, were performed with at least three independent biological replicates.

## Author Contributions

Conceptualization and supervision: X.L., J.T., Y.Z., and Z.M.L. Methodology: X.L., L.Y.C., P.P.L., and J.T. Investigation: X.L., N.N.Z., Z.X.B., L.Y.C., and P.P.L. Data curation: X.L. Visualization: X.L., N.N.Z., and B.B.G. Funding acquisition: X.L., N.N.Z., L.Y.C., Z.M.L., and J.T. Writing the original draft: X.L. Writing the review and editing: X.L., J.T., Z.M.L., and Y.Z.

## Funding

The study was funded by the National Natural Science Foundation of China (Grant Number: 82560293), the Science Project of Science and Technology Department of Jiangxi Province (Grant Number: 20232BAB216024, 20252BAC240538), Wu JiePing Medical foundation (Grant Number: 320.6750.2023‐06‐93), Provincial and Ministerial Co‐constructed Young Talents Project of Henan Medical Science and Technology Tackling Plan (Grant Number: SBGJ202103093), Key Science and Technology Innovation Project of the Jiangxi Provincial Health Commission (Grant Number: 2026ZD008).

## Conflicts of Interest

The authors declare no conflicts of interest.

## Supporting information




**Supporting File 1**: advs77155‐sup‐0001‐SuppMat.xlsx.

**Supporting File 2**: advs77155‐sup‐0002‐SuppMat.docx.

## Data Availability

All data needed to evaluate the conclusions in the paper are present in the paper and/or the Supporting data. RNA‐seq of purified SG cores, Ribosome profiling sequencing, and Single‐cell RNA‐seq data were deposited in the China National Center for Bioinformation database (PRJCA055834). Additional data are available from the corresponding authors upon reasonable request.
