# Supplementary material for: BOULE is Essential for the Dynamic Disassembly of Heat Shock Granules in Male Germ Cells
Source: Adv Sci (Weinh). 2026 Aug 13:e77155. Online ahead of print. doi: 10.1002/advs.77155 (PMC13472119; doi:10.1002/advs.77155)
Supplement: Supplementary file 1 [file ADVS-9999-e77155-s001.docx]

**Supplementary Figures**

**Fig. S1. Protein-protein interaction network analysis.** The network was constructed using 459 germ cell-specific heat shock granule proteins via the STRING database, with the minimum required interaction score set to high confidence (0.700).

**Fig. S2. Formation of heat shock granules in seminiferous tubules at different stages.** Testis tissue was paraffin-embedded after 30 minutes of treatment under room temperature (RT) or 42°C heat shock, followed by sectioning and immunofluorescence co-staining. DAZL is shown in red, PABPC1 in green, and DAPI in blue. Scale bar: 20 µm.

**Fig. S3. Heat shock granules in seminiferous tubules at different stages.** Testis sections from mice treated under room temperature or 42°C heat shock for 30 minutes were co-stained by immunofluorescence. DAZL, G3BP1, and nuclei are shown in red, green, and blue (DAPI), respectively. Scale bar: 20 µm.

**Fig. S4. Analysis of heat shock granule formation in germ cells of seminiferous tubules at different stages.** Testis sections from mice treated under normal temperature or 42°C heat shock for 30 minutes were subjected to immunofluorescence co-staining. DAZL, BOULE, and nuclei are shown in red, green, and blue (DAPI), respectively. Scale bar: 20 µm.

**Fig. S5. Analysis of the dynamic disassembly of heat stress granules in testicular germ cells.** Mouse testes were subjected to heat shock at 42°C for 30 minutes, followed by recovery for 0.5, 1, 1.5, 2, and 2.5 hours. Testis sections were co-stained by immunofluorescence for DAZL (red), BOULE (green), and DAPI (blue). Scale bar: 10 µm.

**Fig. S6. Dynamic changes of heat stress granules in germ cells within seminiferous tubules at different phases of spermatogenesis across various recovery time points.** Testis sections from untreated (room temperature) and heat-shocked (42°C for 30 minutes) mice, as well as from mice allowed to recover for 0.5, 1, 1.5, 2, and 2.5 hours after heat shock, were subjected to immunofluorescence co-staining for DAZL (red), G3BP1 (green), and DAPI (blue). Scale bar: 10 µm.

**Fig. S7. Analysis of ribosome profiling data across control, heat shock, and recovery conditions.** (A) Analysis of ribosome‑protected fragments (RPFs) performed with riboWaltz to assess the three‑nucleotide periodicity in control, heat shock (HS), and heat‑shock recovery (HSR) samples. (B) Bar graph showing the number of genes with detectable ribosome footprints in each experimental group. (C) Bar graph illustrating the distribution of RPFs across the 5′ UTR, coding sequence (CDS), 3′ UTR and intron of protein‑coding genes in control, HS, and HSR groups. (D) Statistical analysis of gene coverage across experimental groups. The coverage value represents the ratio of bases in the coding region covered by mapped reads to the total number of bases in that coding region. (E-G) Heatmaps of differentially expressed genes (DEGs) identified across conditions. Expression values were normalized by z-score transformation and subjected to hierarchical clustering to visualize expression patterns in Control, HS, and HSR groups.

**Fig. S8. Transcriptome analysis of testes under control, heat shock, and heat shock recovery conditions.** (A) Pearson correlation coefficient analysis among samples from room temperature (control), 42°C heat shock (HS), and 150‑minute post‑heat‑shock recovery (HSR) groups. (B) Multi‑group volcano plots displaying differentially expressed genes between the indicated groups. Genes with a fold‑change > 2 and P < 0.05 were considered significant. The number of DEGs is annotated on each plot. C, control; HS, heat shock; HSR, heat shock recovery. (C) Bubble plot of the top enriched GO terms for DEGs between control and HS groups. The 20 most significantly enriched pathways (ranked by P‑value) are shown on the right. (D) Top 20 enriched GO pathways for DEGs between the HSR and HS groups. (E) Top 20 enriched GO pathways for DEGs between the control and HSR groups.

**Fig. S9. G3BP1 ubiquitination levels are significantly reduced in *Boule^-/-^* mouse testes.** (A) Box plot showing the abundance of G3BP1 ubiquitination, as quantified by ubiquitinated proteome analysis, in wild-type (WT) and *Boule^-/^*^-^ testes under control, heat shock (HS), and HS recovery conditions. (B) Comparative analysis of G3BP1 ubiquitination levels between WT and *Boule^-/^*^-^ testes under different treatment conditions. (C) Immunoprecipitation detection of G3BP1 interaction with ubiquitinated proteins in WT heat-shocked testes. (D) Western blot detection of TRIM27 expression in HEK293T cells after knockdown of TRIM27 using independent shRNAs (shTRIM27-1 and shTRIM27-2), with α-TUBULIN as a loading control.

**Fig. S10. Single-cell transcriptome analysis of testes from WT and *Boule^-/-^* mice.**
(A) UMAP plot showing 12 distinct cell clusters identified in *Boule^-/-^* testes, with cell type annotations indicated. (B) Multi-group volcano plots illustrating differentially expressed genes (DEGs) between *Boule^-/-^* and WT testes. Genes with a fold change > 2 and P<0.05 were considered statistically significant. The number of DEGs is annotated on each plot.
